# Two extracellular α-arabinofuranosidases are required for cereal-derived arabinoxylan metabolism by *Bifidobacterium longum* subsp. *longum*

**DOI:** 10.1080/19490976.2024.2353229

**Published:** 2024-05-16

**Authors:** Lisa Friess, Francesca Bottacini, Fionnuala M. McAuliffe, Ian J. O’Neill, Paul D. Cotter, Ciaran Lee, Jose Munoz-Munoz, Douwe van Sinderen

**Affiliations:** a APC Microbiome Ireland, University College Cork, Cork, Ireland; b School of Microbiology, University College Cork, Cork, Ireland; c Biological Sciences, Munster Technological University, Cork, Ireland; d UCD Perinatal Research Centre, School of Medicine, University College Dublin, Dublin, Ireland; e APC Microbiome Ireland, Teagasc Food Research Centre, Cork, Ireland; f School of Biochemistry and Cell Biology, University College Cork, Cork, Ireland; g Department of Applied Sciences, Northumbria University, Newcastle Upon Tyne, UK

**Keywords:** Dietary fiber, prebiotic, bifidobacterial, probiotic, gut microbiota

## Abstract

Members of the genus *Bifidobacterium* are commonly found in the human gut and are known to utilize complex carbohydrates that are indigestible by the human host. Members of the *Bifidobacterium longum* subsp. *longum* taxon can metabolize various plant-derived carbohydrates common to the human diet. To metabolize such polysaccharides, which include arabinoxylan, bifidobacteria need to encode appropriate carbohydrate-active enzymes in their genome. In the current study, we describe two GH43 family enzymes, denoted here as AxuA and AxuB, which are encoded by *B. longum* subsp. *longum* NCIMB 8809 and are shown to be required for cereal-derived arabinoxylan metabolism by this strain. Based on the observed hydrolytic activity of AxuA and AxuB, assessed by employing various synthetic and natural substrates, and based on *in silico* analyses, it is proposed that both AxuA and AxuB represent extracellular α-L-arabinofuranosidases with distinct substrate preferences. The variable presence of the *axuA* and *axuB* genes and other genes previously described to be involved in the metabolism of arabinose-containing glycans can in the majority cases explain the (in)ability of individual *B. longum* subsp. *longum* strains to grow on cereal-derived arabinoxylans and arabinan.

## Introduction

Members of the genus *Bifidobacterium* are Gram-positive bacteria that are commonly found in the gut of mammals, birds, and insects.^1^ They are among the first microorganisms to colonize the human gastrointestinal tract (GIT), where they are particularly abundant during host infancy, before decreasing in relative abundance throughout adolescence and adulthood of their human host.^2^ The gut microbiota composition varies with host age, including the predominant species of bifidobacteria present, which may be attributable to the influence of environmental factors, in particular host diet and the, therefore, resulting differences in available dietary carbohydrates at varying stages of life.^3^ Carbohydrate availability is believed to be closely correlated with bacterial prevalence and abundance in the human intestine because the diet- and/or host-derived glycans can only be accessed by bacteria if they possess the corresponding carbohydrate-active enzymes.^4^ Accordingly, bifidobacteria encode a wide range of carbohydrate-hydrolyzing enzymes, making them highly competitive among other saccharolytic components of the microbial community in the human gut.^5^

Based on fecal sample analysis, *Bifidobacterium longum* appears to be one of the most prevalent bacterial species present in the human colon.^6^ Three different subspecies have been found in the human gut: (i) *B. longum* subsp. *infantis* which is among the first gut colonizers due to its ability to utilize human milk oligosaccharides, (ii) *B. longum* subsp. *iuvenis* which is also able to utilize HMOs in the infant gut,^7^ and (iii) *B. longum* subsp. *longum*, commonly isolated from both infants and adults. The latter subspecies has been shown to utilize various plant-derived oligo- and polysaccharides, which are common to the adult human diet and are typically indigestible by the host.^8^

The fermentative glycan metabolism performed by bifidobacteria predominantly produces acetate and lactate as end products, which are believed to elicit direct and indirect health benefits to the human host. For example, it has been demonstrated in a murine model that the released acetate by *B. longum* subsp. *longum* inhibits epithelial apoptosis and/or exerts an anti-inflammatory effect, therefore decreasing the pathogenic effects of the Shiga toxin produced by *Escherichia coli* O157:H7.^9^ Due to their purported beneficial or probiotic activities, bifidobacteria, including *B. longum* strains, have been incorporated in various commercially available functional foods.^10^

Plant-derived carbohydrates include hemicellulose and dietary fibers, which are found in the cell wall of cereal grains, examples of which are arabinoxylan (AX), arabinogalactan (AG) and arabinan.^11,12^ AX and AX-derived arabinoxylan oligosaccharides (AXOS) consist of a linear xylan backbone composed of β-1,4-linked xylose moieties, which may be decorated by α-1,2- and/or α-1,3-linked arabinose residues.^11^ Due to its abundance in the cereal cell wall, AX can be extracted from several different sources, including wheat, rye, oat, rice, barley, and corn. Depending on the source and extraction procedure, AX may have varying levels of branching and contain different substitutions, like D-galactose or ferulic acid, causing for example corn-derived AX to be much more complex in structure when compared to wheat AX.^13–15^

Due to the narrow substrate specificity of enzymes, multiple enzymes are typically required to utilize and fully degrade complex polysaccharides. Specifically for AX, exo-α-L-arabinofuranosidases (classified into glycoside hydrase (GH) families GH3, GH43, GH51, GH54 or GH62),^16–18^ are required for the hydrolysis of the α-1,2 and α-1,3 bonds in order to release L-arabinose substitutions from the xylan backbone. Degradation of the xylan backbone in turn requires an (extracellular) endo-1,4-β-xylanase (classified into the GH10, GH11, GH30, GH43, GH51 or GH54 families)^16,19^ which cleaves the β-1,4 bonds that connect the ᴅ-xylose residues to produce xylo-oligosaccharide (XOS).^20^ Due to multiple, but varying numbers of, α-L-arabinofuranosidases predicted to be encoded by different *B. longum* strains, it is possible that certain enzymes show overlapping substrate specificity and that different strains are able to grow on different arabinose-containing glycans.^21^ An arabinan utilization gene cluster (termed here as the *abu* cluster) found in *B. longum* subsp. *longum* JCM 1217, encoding five GH43 enzymes, has experimentally been proven to be active on arabinan and AG.^12^ Enzymes have been characterized to be active on the α-1,3-ara*f* substitution of AG (BlArafA), the α-1,2 and α-1,3 substitutions of arabinan (BlArafC) and the α-1,5 bonds in the arabinan backbone (BlArafB). Also, it has been shown by *in vitro* experiments that enzymes in this cluster are active on AX: BlArafE, active on the α-1,3-ara*f* substitution, and BlArafD, containing two active sites and able to hydrolyze the α-1,2-ara*f* sidechain, as well as the α-1,5 bonds in the arabinan backbone.^22^ The cluster further encodes predicted ABC-type transporter proteins and a LacI transcriptional regulator.^12^ This gene cluster is either fully or partially conserved or absent among individual members of the *B. longum* subsp. *longum* taxon.^12^ For example, *B. longum* subsp. *longum* NCIMB 8809 retains a gene encoding a predicted extracellular GH43 enzyme, homologous to BlArafA in *B. longum* JCM1217 as well as its transcriptional regulator and sugar transporters, however it lacks homologues of four (BlArafB to BlArafE) GH43-encoding genes of this cluster. Interestingly, the genome of *B. longum* NCIMB 8809 contains a different GH43-encoding gene cluster, called the alpha arabino-oligosaccharide utilization (*aau*) locus with five distinct GH43 enzymes,^4,8,23^ which is also highly conserved across the species (though it is absent in *B. longum* subsp. *longum* JCM1217). By *in silico* analysis this cluster has been hypothesized to be involved in either AX or AOS metabolism, though experimental proof is currently lacking to support this notion.^23^ The *aau* cluster also encodes a LacI transcription factor, ABC transporter proteins and an experimentally verified esterase, shown to cleave hydroxycinnamic acids from arabinose.^24^

In the current study a third, two-gene, cluster named here as *axu* (for arabinoxylan utilisation), is described for *B. longum* subsp. *longum* NCIMB 8809, encoding two extracellular GH43 enzymes (AxuA and AxuB) which are shown to be responsible for growth of this strain on AX. Using enzymatic analysis, mutant characterization and growth experiments we demonstrate their specific role in cereal-based AX metabolism in two members of the *B. longum* subsp. *longum* taxon.

## Materials and methods

### Growth media and bacterial cultivation conditions

Strains and plasmids are listed in Table S1. *Escherichia coli* strains were routinely cultivated in Luria-Bertani (LB) broth^25^ at 37°C with agitation at 180 rpm. *B. longum* subsp. *longum* strains were routinely cultured in modified de Man Rogosa and Sharpe medium excluding a carbohydrate source (referred to here as mMRS; see reference^26^  + 0.06% cysteine-HCl (w/v; Sigma-Aldrich, Steinheim, Germany) + 1% lactose (w/v; (Sigma-Aldrich)) and incubated anaerobically in a modular atmosphere-controlled system (Davidson and Hardy, Belfast, United Kingdom) at 37°C. Where appropriate, a given growth medium was supplemented with kanamycin (Km; 50 μg ml^−1^ for *E. coli*), erythromycin (EM; 250 μg ml^−1^ for *E. coli*, 100 μg ml^−1^ for *B. longum*) or tetracycline (TC; 10 μg ml^−1^ for *E. coli*, 5 μg ml^−1^ for *B. longum*). Carbohydrate utilization by bifidobacterial strains was examined in mMRS medium supplemented with cysteine-HCl (0.06% w/v; (Sigma-Aldrich)) and a particular carbohydrate (0.5%, wt/v). Assessed carbohydrates were lactose, xylose (Sigma-Aldrich), xylan from beechwood (Megazyme, Wicklow, Ireland), xylo-oligosaccharides (XOS; Shandong Longlive Bio-Technology Co., Japan), arabinoxylan from rye (AXR; Megazyme), arabinoxylan from wheat (AXW; Megazyme), arabinan (Megazyme), arabinose (Sigma-Aldrich, Steinheim, Germany), galactan (Megazyme), larch arabinogalactan (Megazyme). To determine bacterial growth profiles and final optical densities, 5 ml freshly prepared mMRS medium, including a particular carbohydrate (see above), was inoculated with 50 μl (1% inoculum; OD_600 nm_ ~1) of a particular *B. longum* subsp. *longum* overnight culture in mMRS + 0.06% cysteine-HCl (w/v) + 0.5% lactose (w/v). Uninoculated mMRS medium was used as a negative control. Cultures were incubated anaerobically at 37°C and the optical density at 600 nm (OD_600 nm_) was determined manually after 24 hours. Assays were repeated in triplicate.

### Transcriptome analysis by RNAseq

*B. longum* subsp. *longum* NCIMB 8809 was grown overnight in mMRS (15) + 0.06% cysteine-HCl +1% lactose, and then subcultured in mMRS + 0.06% cysteine-HCl +0.5% lactose, arabinose or AXR until an OD_600 nm_ of approximately 0.5 was reached. Cells were harvested from 3 mL of culture by centrifugation and then resuspended in 300 μL RNA shields (Zymo Research, Orange, CA, USA). The resulting cell suspensions were sent to BaseClear (Leiden, The Netherlands). RNA isolation by Baseclear (Leiden, The Netherlands) with ZymoBIOMICS™ RNA Miniprep Kit (Zymo) and sequencing was performed on an Novaseq 6000 (Illumina) with paired-end 150 cycles. Following sequencing, the resulting reads were depleted of adapters, quality filtered (with overall quality, quality window and length filters) and aligned to the *B. longum* subsp. *longum* NCIMB8809 reference genome (genomic features model GTF file^27^) through bowtie2 aligner.^28^ The alignment of SAM files was further processed using Samtools to obtain BAM files necessary to obtain matrices with read counts per gene (normalized by gene length). Differential gene expression (DGE) analysis was performed using the R statistical platform and the DESeq2 package available as part of the Bioconductor release (v.3.9). As a pre-processing step, rows with zero counts (unmapped genes) were discarded from the count matrices. Differential expression analysis was performed on the count matrices using the DESeq function in DESeq2. Genes with an FDR-adjusted p-value of < 0.05 and a log2-fold change of > 3 were considered significantly upregulated.

### Genotype analysis

BLASTP- and BLASTN-mediated all-against-all searches^29^ were performed for 25 *B. longum* subsp. *longum* genome sequences and two publicly available *B. longum* subsp. *longum* genomes (NCIMB 8809 and JCM 1217), in order to determine the presence or absence of genes encompassing the *abu*, *aau* and *axu* gene clusters, as well as several genes encoding predicted extracellular GH43 enzymes.

### GTM associations

Following completion of the growth assessment of *B. longum* strains, an *in silico* genotype/phenotype gene-trait matching (GTM) exercise was performed, correlating the presence/absence of genes obtained from comparative analysis with an observed phenotype. The analysis was performed on a set of genes corresponding to growth ability on galactan (corresponding to the *gala* gene), on arabinan (corresponding to the presence of the *BlArafB* and/or *BlArafC* genes) and on arabinoxylan (genes encoding various predicted extracellular GH43 enzymes).

### His-tagged AxuB and AxuA overexpression and purification

For overexpression and purification of His-tagged AxuA and AxuB (for this purpose designated as AxuA_His_ and AxuB_His_, respectively), plasmids pET28b:1599 and pET28b:1600 were generated as follows. The genes corresponding to locus tags B8809_1599 without the transmembrane domains (corresponding to the gene designated here as *axuB;* from codon 58 to 931 or position 1,984,332 to 1,986,950 of the deposited genome sequence [accession number: PRJNA287223]), and B8809_1600 without the secretion signal and transmembrane domains (corresponding gene designated here as *axuA*, from codon 45 to 823 or position 1,987,431 to 1,989,764) were amplified by PCR (Q5 High-Fidelity DNA polymerase; NEB; primers can be found in Tab. S2) using genomic DNA extracted from *B. longum* subsp. *longum* NCIMB 8809 as a template and cloned into pET28b, utilizing its N-terminal His-tag (Novagen), using EcoRI and NotI for *axuB*, and NheI and NotI for *axuA*. *E. coli* 10-beta cells (NewEngland Biolabs, Ipswich, MA, US) were used as cloning host,^30^ before transferring the individual recombinant plasmids into BL21 (DE3) cells. To validate their genetic integrity all constructs were verified by DNA sequencing (Plasmidsaurus, Eugene, OR, US).

To achieve protein overexpression, 100 mL NZY media (1% (v/v); NZYtech, Lisbon, Portugal) was inoculated with 1% of a particular *E. coli* strain and incubated for 24 h at 24°C, 300 rpm. Cells were harvested by centrifugation before resuspending the cells in a lysis buffer (50 mM Tris-Base (Fisher Bioreagents, US), 300 mM NaCl (Fisher Bioreagents), 50 mM CaCl_2_ (Fisher Bioreagents), 10 mM imidazole (Sigma-Aldrich)); cell lysis was then achieved employing a mini-beadbeater (BiospecProducts, USA; 3 times 30 sec alternating with 30 sec incubations at 4°C). Protein purification was performed using a TALON Superflow column (Cytiva, Marlborough, MA, USA), using elution buffers with imidazole concentrations ranging from 20 mM to 500 mM. To analyze and estimate the molecular weight of the recombinant AxuB_His_ and AxuA_His_ proteins, sodium dodecyl sulfate-polyacrylamide gel electrophoresis (SDS-PAGE) was performed as previously described^31^ including Colour Prestained Protein Standard, Broad Range (10–250 kDa, NEB), and gels were stained using Coomassie Brilliant Blue. Elution fractions of interest were concentrated and dialyzed using Amicon® Ultra Filters Merck Millipore (Merck, Darmstadt, Germany), in 20 mM sodium phosphate buffer pH7. The concentration of the protein was estimated using the Qubit® Fluorometer (Thermo Scientific, Gloucester, UK), and the corresponding protein assay kit (Thermo Scientific).

### Enzyme assays

Enzyme assays using the synthetic p-Np-substrates: p-Np-α-L-arabinofuranose (Carbosynth, Berkshire, UK), p-Np-α-L-arabinopyranose (Carbosynth), p-NP-β-D-xylopyranose (Carbosynth) were performed at 37°C in 20 mM potassium phosphate buffer (pH7) with 2, 4, 5, 7.5 and 10 mM of the substrate and 0.5 µM of AxuB_His_ or 0.1 µM of AxuA_His_. The enzymatic activity was determined quantitatively by spectrometer measurement (OD_405 nm_) every 30 sec for 5 min.

Enzymatic assays on plant-based or synthetic substrates were performed in 20 mM potassium phosphate buffer (pH7) at 37°C using the following substrates (Megazyme): 3^2^-α-L-arabinofuranosyl-arabinotriose (AA3A; further structure description can be found in Tab. S3), 2,^2^ 3^2^-α-L-arabinofuranosyl-arabinotriose (AA23A) + 3^3^-α-L-arabinofuranosyl-arabinotetraose (AA3AA), 2^3^-α-L-arabinofuranosyl-xylotriose (A2XX), 2,^3^ 3^3^-di-α-L-arabinofuranosyl-xylotriose (A23XX) and 3^2^-α-L-Arabinofuranosyl-xylobiose (A3X) at a concentration of 0.5, 1 and 2 mg/mL and 0.5 µM of AxuB_His_ or 0.1 µM of AxuA_His_. Monomeric L-arabinose release was measured using the L-Arabinose/D-Galactose Assay Kit (Megazyme) every 30 sec for 5 min. Enzymatic reactions were carried out in triplicate.

### HPAEC-PAD analysis

For HPAEC-PAD analysis, a Thermo Scientific Dionex (Sunnyvale, CA) ICS-3000 system was used. Carbohydrate fractions from the above-mentioned hydrolysis assays (200 μl aliquots) were separated on a CarboPac PA1 analytical exchange column (dimensions, 250 mm by 4 mm) with a CarboPac PA1 guard column (dimensions, 50 mm by 4 mm) and visualized by a pulsed electrochemical detector (ED40) in PAD mode (Thermo Scientific Dionex). Elution was performed with 100 mM of potassium hydroxide (KOH) at a constant flow rate of 0.063 ml/min at 30°C for 30 min. Chromatographic profiles of each of the tested carbohydrates, as well as their putative breakdown products (where available), were used as reference standards for identification during quantitative analysis of the results of the glycan breakdown by AxuB_His_ and AxuA_His_. Chromeleon software (version 7.3; Dionex Corporation) was used for the integration and evaluation of the chromatograms obtained.

### Generation of insertion mutations in *axuB* or *axuA* of *B.* *longum* subsp. *longum* NCIMB 8809

Internal 846 bp fragments of *axuB* (from codon 68 to 349 out of 958 or from position 1,986,075 to 1,986,920 of the deposited genome sequence (accession number: PRJNA287223)) or 468 bp fragment of *axuA* (from codon 11 to 167 out of 850 or from position 1,989,397 to 1,989,864) were amplified by PCR (primers can be found in Tab. S2) using *B. longum* subsp. *longum* NCIMB 8809 chromosomal DNA as template. The generated PCR products were ligated to pFREM2 (see supplementary: this plasmid is a modified version of pFREM28 with removed restriction modification motifs for *B. longum*), using XbaI and NcoI restriction sites, and introduced into *E. coli* EC101 as cloning host.^30^ *E. coli* EC101 derivatives containing the expected recombinant pFREM2 constructs were selected on LB agar containing erythromycin. *B. longum* subsp. *longum* NCIMB 8809 was transformed as previously described with minor adaptations.^32–36^ Briefly, a single colony was grown in MRS broth (MRS media, Difco, Fisher Scientific, USA) at 37°C for 16 hours and twice subculture in MRS broth with the addition of 7% sucrose (Fisher Scientific, USA) and 34 μg/ml Iron(II) Sulfate Heptahydrate (Fisher Scientific, USA) and grown until an OD_600 nm_ of 0.9–1.1 was reached, before 10% inoculation of 50 mL of MRS broth reconstituted from individual components, modified with 1% lactose (Sigma-Aldrich, USA) and 7% sucrose and an additional 20 mM NaCl (Fisher Scientific, USA) and grown until the culture reached an OD_600 nm_ of 0.35–0.5. Cells were then harvested by centrifugation (10 min, 4,500 × *g*) and washed 3 times with 2 mL of 0.5 M sucrose in 1 mM citrate buffer (transferred into 2 mL tubes; 1 min 15,000 *x g*) and resuspended in 200 μL 0.5 M sucrose in 1 mM citrate buffer (transfer steps were carried out in an anaerobic chamber). Competent cells and added plasmid DNA were incubated on ice for 30 min (in the anaerobic chamber) prior to electroporation (2.5 V, 20 kW, 300Ω). Cells were recovered in MRS broth modified with 100 ng/mL CaCl_2_ (w/v) and 50 ng/mL (w/v) 1,4-dihydroxy-2-naphthoic acid (DHNA;^37^ Sigma-Aldrich, USA) for 3 hours at 39°C, plated on RCA with 100 ng/mL CaCl_2_ (w/v) and 50 ng/mL (w/v) DHNA and appropriate antibiotic at 39°C for 48 hours.

To validate their genetic integrity all insertion mutants were verified by PCR (OneTaq 2×MM; NEB) and their genetic integrity was subsequently corroborated by DNA sequencing of the PCR product (Genewiz, Germany).

### Construction of complementation and expression vectors

For phenotypic complementation of the insertion mutants and expression of *axuB* and *axuA* in MM0464, *axuA* together with a 364 bp upstream region (which contains its native promoter as based on the RNAseq data) and *axuB* together with an artificial promoter were amplified by PCR (Q5 High-Fidelity DNA polymerase; NEB; primers can be found in Tab. S2) using *B. longum* subsp. *longum* NCIMB 8809 chromosomal DNA as template. The PCR products generated were ligated to pBM5 (see supplementary), using ApaI and XhoI restriction sites, and introduced into *E. coli* DH5α as cloning host,^30^ before transferring the individual recombinant plasmids into EC101 cells harboring pNZEM.

To validate their genetic integrity all constructs were verified by DNA sequencing (Plamidsaurus, Eugene, OR, USA). The plasmids were extracted using GeneJET Plasmid Miniprep Kit (Thermo Fisher Scientific, Waltham, MA, USA) and transformed into *B. longum* subsp. *longum* NCIMB 8809 as described above.

## Results

### Growth abilities of *B.* *longum* subsp. *longum* strains on plant-derived glycans

**Figure 1.**
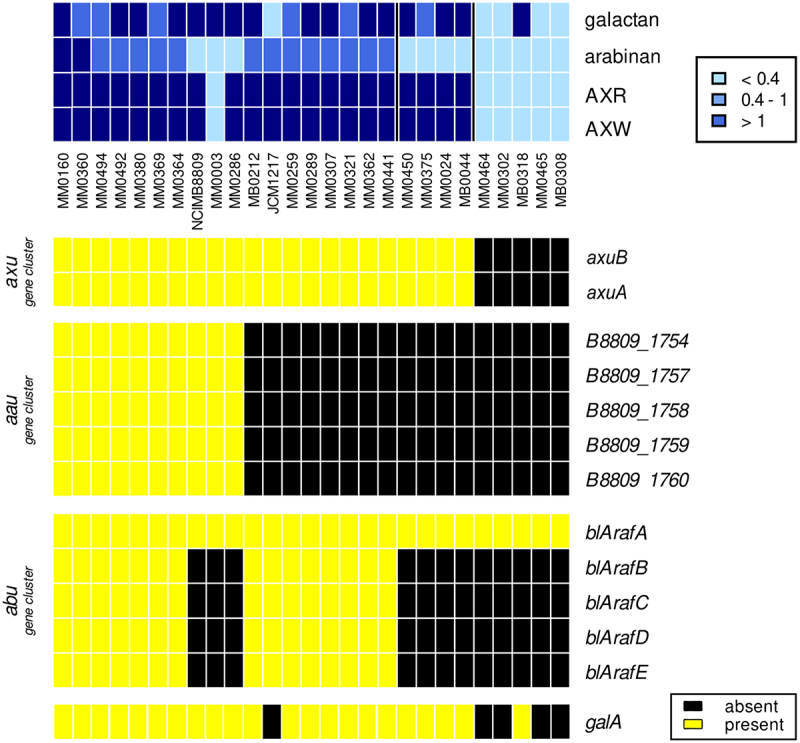
Growth profiles after 24 hours of incubation (top panel) and corresponding gene map (bottom panel) of *B. longum* subsp. *longum* strains *B. longum* subsp. *longum* strains (names indicated below the top panel) were cultivated for 24 h in mMRS containing particular carbohydrates (indicated on the right-hand margin in the top panel) and growth is indicated in blue color coding based on OD readings as indicated. Bottom panel displays the presence/absence of genes involved in the metabolism of plant glycans in the corresponding genomes of the *B. longum* strains, where yellow indicates presence and black absence. The cutoff presence or absence of the gene/enzyme was set to E-value of 0.0001, with at least 50% identity across at least 50% of either protein sequence.

25 *B. longum* subsp. *longum* strains, which had previously been isolated from fecal samples from mother-baby dyads as part of the Microbe Mom study,^38,39^ and two obtained from public strain collections were cultivated on mMRS supplemented with a range of different plant-derived glycans (see Materials and Methods), in order to assess their growth abilities based on the use of such carbohydrates as the sole carbon and energy source (Figure 1). All tested *B. longum* subsp. *longum* strains were shown to exhibit substantial growth (OD_600 nm_ of > 0.4 after 24 h cultivation) on lactose, arabinose, and xylose. None of the studied strains was shown to elicit growth (OD_600 nm_ <0.4 after 24 h cultivation) in a medium containing xylan from beechwood or larch AG as the sole carbon source. For arabinan, galactan, arbinoxylan from rye (AXR), and from wheat (AXW) several strains did exhibit clear ability to metabolize these carbohydrates, while other strains were unable to grow (Figure 1).

### Gene-trait matching

The 25 strains obtained from the Microbe Mom study were genome sequenced (see supplementary; Tab. S4 for details). In order to identify genotype-phenotype connections for particular glycan substrates on which only a proportion of the tested *B. longum* subsp. *longum* strains achieved significant growth, a gene-trait matching exercise was performed (see supplementary). Previously, GalA has been described as an extracellular endo-galactanase releasing galactotriose from type I galactans,^40^ together with an ABC transport system, encoded by *galBCDE*.^41^ Our gene-trait matching results show that all strains that lack *galA* are unable to grow to any appreciable degree on galactan, and conversely all strains harboring this gene are able to grow on this glycan (Figure 1).

**Figure 2.**
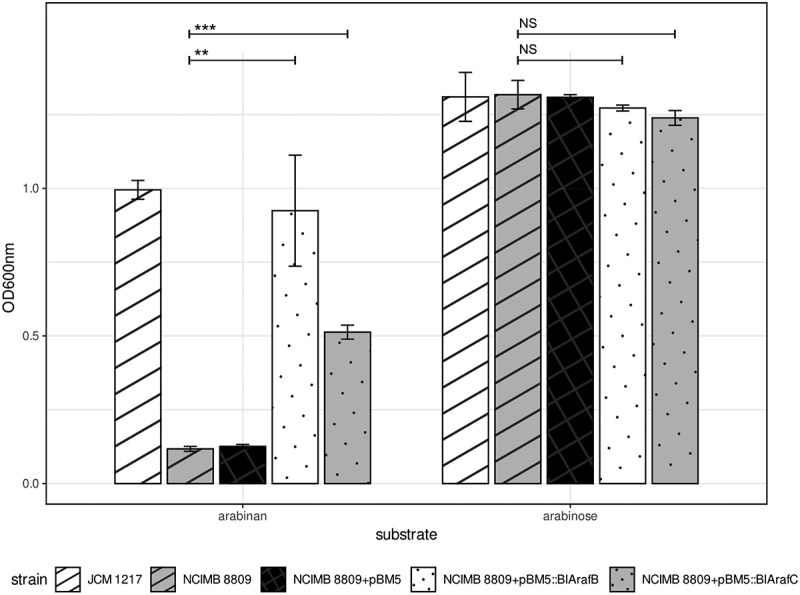
Growth abilities of *B. longum* subsp. *longum* NCIMB 8809 and derivatives with genes from the *abu* gene clusterOd600nm after 24 hours anaerobic growth in mMRS + 0.5% (w/v) arabinan or arabinose. Asterisks represent a significant difference (*p* ≤ 0.001***, *p* ≤ 0.01**) and NS indicates no significant difference (*p* ≥ 0.05).

Concordantly, 15 strains out of the 27 assessed strains were able to grow on arabinan as the sole carbon source and harbor the (full) *abu* gene cluster, while the 12 remaining strains without the *abu* gene cluster were unable to grow on arabinan (Figure 1). This confirms a gene-trait connection between the *abu* gene cluster and the ability to utilize arabinan.^12^ To further validate this, *blArafB* and *blArafC* were cloned into a *B. longum* subsp. *longum* strain which lacks this cluster, and which is not able to utilize arabinan (see supplementary). The introduction of either of the two genes encoding α-L-arabinofuranosidases of the *abu* gene cluster into *B. longum* subsp. *longum* NCIMB 8809 was shown to result in either partial restoration of growth (in the case of *BlArafC* introduction; active on the α-1,2 and α-1,3 substitutions of arabinan) or near identical growth (*BlArafB* introduction; active on α-1,5 bonds in the arabinan backbone) when compared to *B. longum* subsp. *longum* JCM 1217 after 24 h hours (Figure 2).

As mentioned above, the *abu* cluster and/or the *aau* gene cluster have been associated with AX utilization in *B. longum* subsp. *longum*. A total of 21 strains were shown to be able to utilize AXR/AXW as the sole carbon source; however, no apparent gene-trait matching profile was identified when focusing solely on either of these two clusters. Notably, several strains lacking both clusters (Figure 1) were still able to grow in media containing AX as the sole carbon source, an observation indicating that GH43 enzymes other than those encoded by the *abu* and *aau* clusters are required for AX metabolism in such strains.

### Transcriptome analysis to identify genes involved in cereal-derived arabinoxylan metabolism

**Figure 3.**
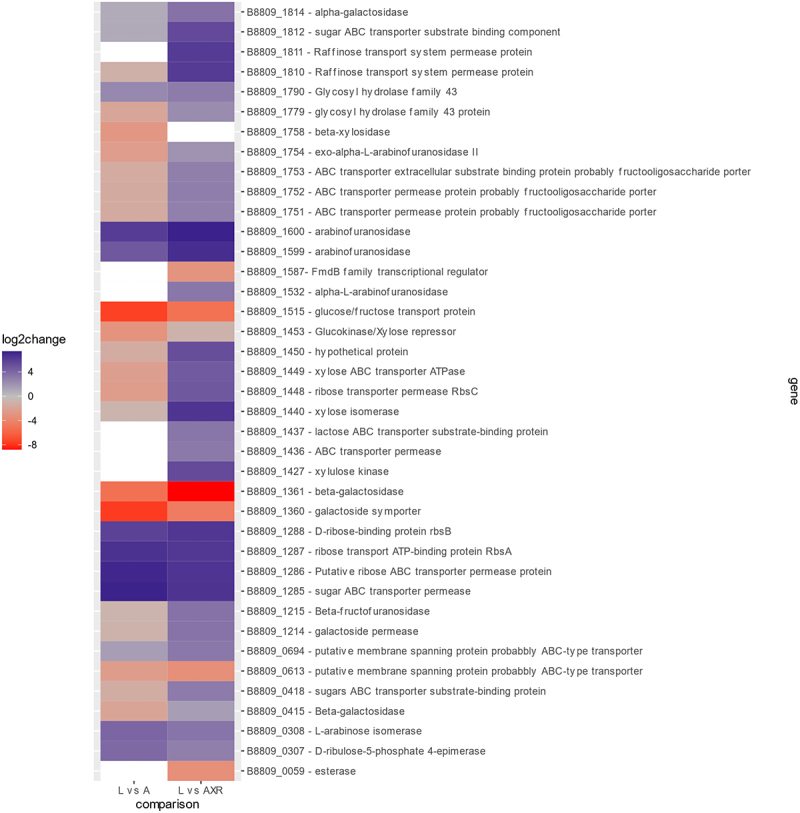
Heatmap of differential expressed genes differential expressed genes (DEGs) in *B. longum* subsp. *longum* NCIMB 8809, with significant levels of change in their degree of log_2_ fold expression, when comparing arabinose (left) or AXR (right) to a lactose control. Red: negative log_2_ fold change (down-regulated), gray: zero log_2_ fold change, blue positive log_2_ fold change (up-regulated), and white no significant change under these conditions.

In order to identify genes involved in AX metabolism an RNAseq analysis was performed in which the transcriptomes generated by *B. longum* subsp. *longum* NCIMB 8809 grown on lactose, arabinose or AXR were compared (see supplementary). Genes whose transcription was shown to be significantly up or downregulated when grown on arabinose or AXR as compared to growth in lactose were identified and represented (Figure 3).

Among the transcriptionally upregulated genes are genes from the *aau* gene locus which were upregulated when growing on AXR compared to lactose, which further indicates that these genes are involved in AXR utilization. These specifically include two GH43 enzymes (B8809_1754/B8809_1758) and three genes predicted to represent an ABC transporter (B8809_1751-B8809_1753). Furthermore, we would expect to observe upregulation of genes predicted to encode extracellular enzymes active on either the arabinose sidechains or the xylan backbone, thus allowing extracellular hydrolysis of the AXR polymer into mono-/oligosaccharides, which are then transported into the cytoplasm to enter metabolic pathways. Five genes fulfilled the above expectations, i.e., not being connected to either the *abu* or *aau* cluster, and representing AXR-upregulated putative extracellular α-L-arabinofuranosidase-encoding genes in *B. longum* subsp. *longum* NCIMB 8809: (i) B8809_1532, encoding a predicted extracellular α-L-arabinofuranosidase, but with no similarity to any studied GH group,^42^ (ii) B8809_1779, a secreted GH43 enzyme with no similarities to characterised enzymes, (iii) B8809_1790, a GH43 enzyme, which shows an 99% identity with the previously characterized BlArafA (BLLJ_1854), which has been shown to cleave α-1,3-arabinose side chains from arabinogalactan,^43^ and (iv) B8809_1599 and (v) B8809_1600, which both are predicted to encode extracellular arabinofuranosidases belonging to the GH43 family.

Furthermore, transcriptional upregulation of genes involved in utilization of the released arabinose was expected. Such genes should be upregulated in cells grown in either AX or arabinose, when compared to cells grown in lactose. Among these arabinose-upregulated genes is a gene cluster of three genes whose products are predicted to form an ABC-type transporter system involved in a pentose sugar uptake (B8809_1285-B8809_1287, the proposed *araFGH* operon.^23^ Also upregulated is the transcription of two adjacent genes corresponding to locus tags B8809_0307 and B8809_0308 AraABD,^44^ which are predicted to be involved in the conversion of arabinose to enter the so-called ‘bifid’ shunt.^45^

Genes for the uptake of xylose/XOS released from the backbone of AX and their utilization might also be upregulated. A gene cluster that is upregulated (consisting of four genes with the locus tags B8809_1436, B8809_1437, B8809_1448 and B8809_1449) is predicted to encode a pentose sugar ABC transporter, which may be responsible for xylose/XOS uptake.

### Gene-trait matching based on genes identified by transcriptome analysis and AX growth

The results of the transcriptome analysis of *B. longum* subsp. *longum* NCIMB 8809 and growth profiles of *B. longum* subsp. *longum* strains on AXR and AXW were combined in a gene-trait matching effort, specifically focussing on the absence/presence of homologous genes encoding predicted extracellular GH43 enzymes in the *B. longum* subsp. *longum* strains and their ability to grow on AXR and AXW. While the absence/presence of (homologues of) B8809_1532, B8809_1779 or B8809_1790 does not seem to be associated with the (in)ability of the corresponding strain to grow on AXR or AXW, the two adjacent genes B8809_1599 and B8809_1600 (which we henceforth refer to as the *axu* cluster for arabinoxylan utilization) encoding predicted α-arabinofuranosidases were considered promising candidates, as the presence of these two genes was shown to correspond to the ability of a strain to utilize AXR and AXW. As can be observed from the results displayed in Figure 1, 23 out of 27 tested strains harbor the genomic *axu* cluster and are able to utilize AXR and AXW as a growth substrate, with the exception of MM0003. Compared to the *axuA* gene of *B. longum* subsp. *longum* NCIMB 8809, the genome of strain MM0003 does indeed harbor an *axuA* homologue (99% identity over 95% of *axuA*), however the 5'-part of this gene and upstream region in strain MM0003 appears to be deleted. This deletion is likely to affect expression and activity of the enzyme, thus explaining the inability of this strain to grow on AXR/AXW. All strains lacking the *axu* gene cluster are unable to grow when either AXR or AXW is the only available carbon source.

### In silico *analysis of the axu cluster*

**Figure 4.**
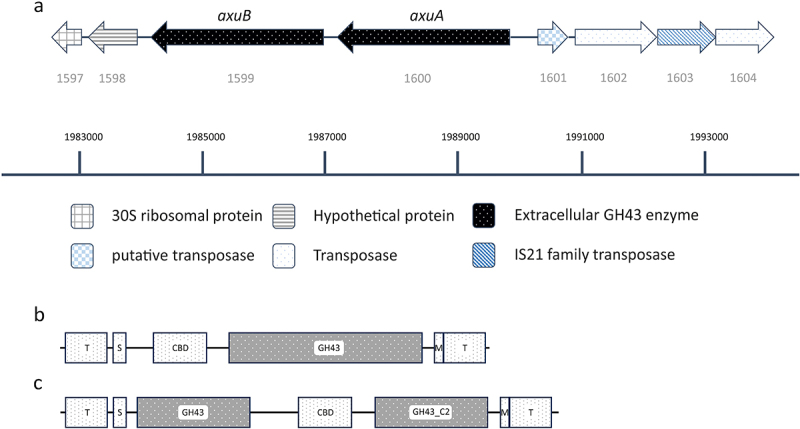
Schematic display of the *axu* gene cluster and neighboring genes in *B. longum* subsp. *longum* NCIMB 8809. (panel A). Illustration of the domains present in AxuA (panel B) and AxuB (panel C), predicted by TMHMM, SignalP, and HMMER; T: transmembrane domain; S: signal peptide; CBD: carbohydrate binding domain; M: sortase motif.

To perform a functional prediction of the two enzymes encoded by the *axu* cluster an analysis of the AxuA and AxuB protein sequences was made (see supplementary). AxuB is predicted to consist of a two-domain GH43_10 extracellular enzyme with an N-terminal transmembrane domain corresponding to its secretion signal, while it also harbors a sortase motif^46^ and hydrophobic membrane-spanning domain at its C-terminal end (Figure 4; Hits gained by SignalP, Hmmer and HHpred are summarized in Table S5). One of the GH43 domains is predicted to be a catalytic domain, while the second is predicted to represent a C-terminal accessory domain.^42^ Sequence alignment with HHpred^47^ predicts that it shares structural similarity with AXHd3 isolated from *Humicola insolens*, which is an α-arabinofuranosidase, and which has been shown to hydrolyze the α-1,3 linkage of double arabinose-substituted xylans.^48^ Interestingly, an association between AX degradation and an *axuB* homologue (99.8% identity) in *B. longum* subsp. *longum* NCC2705 (NCC2705_1544) has previously been made.^21^

The AxuA protein is predicted to be an extracellular enzyme having a single GH43_16 domain with an N-terminal transmembrane domain corresponding to its secretion signal and a sortase motif as well as a hydrophobic membrane-spanning domain at its C-terminal (Figure 4; Hits gained by SignalP, Hmmer and HHpred are summarized in Table S5). Alignments show that it is similar to ARN3 (produced by *Thermotoga petrophila* ^49^ and BsArb43B (produced by *Bacillus subtilis*,^50^ previously described as endo-1,5-alpha-L-arabinanases, also encompassing two carbohydrate-binding domains. Another similarity hit was obtained for *B. subtilis* enzyme BsAXH-m2,3, an enzyme shown to hydrolyze mono-substituted α-1,2 or α-1,3 arabinose residues from a xylose backbone.^51^ A highly homologous gene in B. *longum* subsp. *longum* NCC2705 (XynD; NCC2705_1543) has previously been proposed as an extracellular endoxylanase playing a key role in the hydrolysis of AX as indicated by the upregulation in gene expression determined by quantitative real-time reverse transcription analysis,^52^ although the specific activity of this enzyme was never reported.^21^ XynD was classified as endoxylanase while the presence of a sortase motif indicates its extracellular anchoring to the cell wall.^53^ Notably, a XynD homolog encoded by *Bacillus polymyxa* was shown to act as a xylanase as well as an α-L-arabinofuranosidase.^54^

Unlike the *abu* or *aau* clusters, this *axu* cluster does not harbor or is flanked by genes predicted to be involved in carbohydrate transport or transcriptional regulation and is bordered by genes encoding a hypothetical protein and transposases (Figure 4A).

### Assessment of enzymatic activity

To investigate the enzymatic activity of each of two encoded products of the *axu* gene locus, the two genes were individually cloned (without their encoding signal and membrane-anchoring sequences), allowing cytoplasmic overexpression and subsequent purification of the corresponding protein products (see Materials and Methods). The observed molecular weight of each purified protein as determined by SDS-PAGE analysis, 84 kDa for AxuA_His_ and 100 kDa for AxuB_His_, is in good agreement with the corresponding EXPASY calculated molecular weights (i.e., 89 kDa for AxuA_His_ and 105kDa for AxuB_His_) (SDS-page; Fig. S1). AxuB_His_ and AxuA_His_ were then tested for their hydrolytic activity toward various 4-Nitrophenyl and arabino- and arabinoxylo-oligosaccharides.

Under the conditions employed, AxuA_His_ was shown to exhibit hydrolytic activity toward pNp-L-arabinofuranose (K_cat_/K_m_ values shown in Table 1), but not p-Np-β-D-arabinopyranose or p-Np-β-D-xylopyranose, which indicates the enzyme acts as a α-arabinofuranosidase or at least is able to cleave the 4-nitrophenyl substituent at the anomeric position. AxuB did not show activity against any of the tested pNp-substrates.

**Table 1.** Summary of obtained K_cat_/K_m_ values of AxuA_His_ and AxuB_His._

	AxuB_His_	AxuA_His_
	k_cat_/K_m_ (min^−1^µM^−1^)	
p-Np-α-L-arabinofuranose	*	0.115
p-Np-β-D-arabinopyranose	*	*
p-Np-β-D-xylopyranose	*	*
A2XX	*	0.201
A3X	*	0.084
A23XX	0.00628	*
AA3A	*	*
AA23A + AA3AA	*	*

Kcat/Km values for p-Np- substrates and AXOS for AxuB_His_ and AxuA_His_ measured over 5 min; A2XX: 23-α-L-Arabinofuranosyl-xylotriose, A3X: 32-α-L-Arabinofuranosyl-xylobiose, A23XX: 23,33-di-α-L-Arabinofuranosyl-xylotriose; * activity below detection limit.

**Figure 5.**
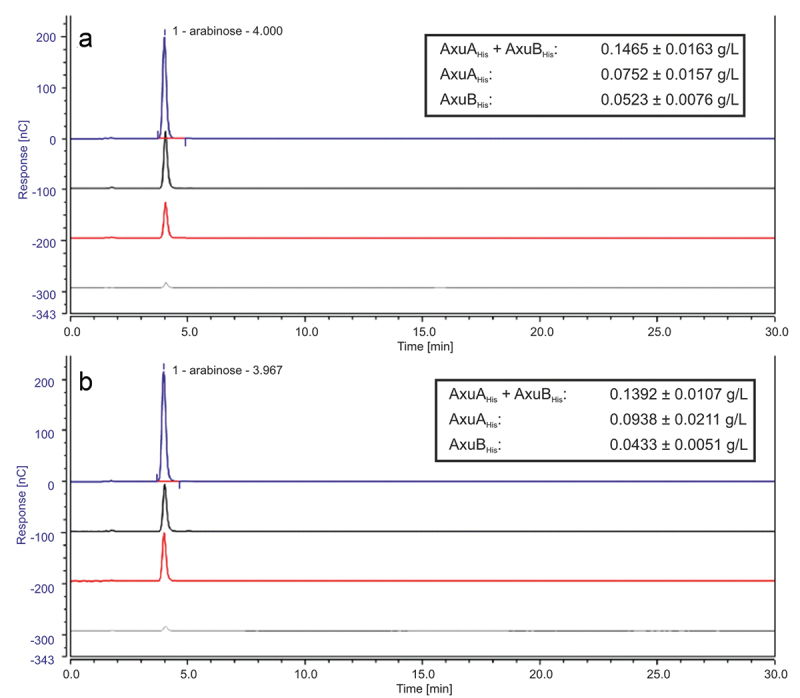
HPAEC-PAD analysis of hydrolysis products of AX incubated with AxuA_His_/AxuB_His–_AXW (panel A), AXR (panel B). From bottom to top: substrate only (gray), with AxuBHis (red), with AxuAHis (black), with both (blue) after 30 min. In box: amount (g/L) of release monomeric L-arabinose of 1 mM enzyme after 30 min of incubation.

To verify the expected hydrolytic activity of AxuA_His_ and AxuB_His_ toward AX, both proteins were further analyzed employing AX isolated from wheat (AXW) or rye (AXR). Both AxuA_His_ and AxuB_His_ were demonstrated to release arabinose from either of the two tested AX preparations, as shown in Figure 5, thus validating the presumed arabinofuranosidase activity of AxuA. AxuB_His_ was shown to release a smaller amount of arabinose when compared to AxuA, and when both enzymes were combined in a reaction the amount of released arabinose is similar to the addition of both enzymes separately for AXR (values in Figure 5A), and higher for AXW (values in Figure 5B). The structural differences in AX and the quantity of double or single arabinose-substitutions appear to result in variations of released L-arabinose. AxuA_His_ was shown to be able to cleave off more arabinose from AXR than from AXW, while conversely AxuB_His_ was shown to release a larger amount of arabinose from AXW than from AXR (Figure 5).

**Figure 6.**
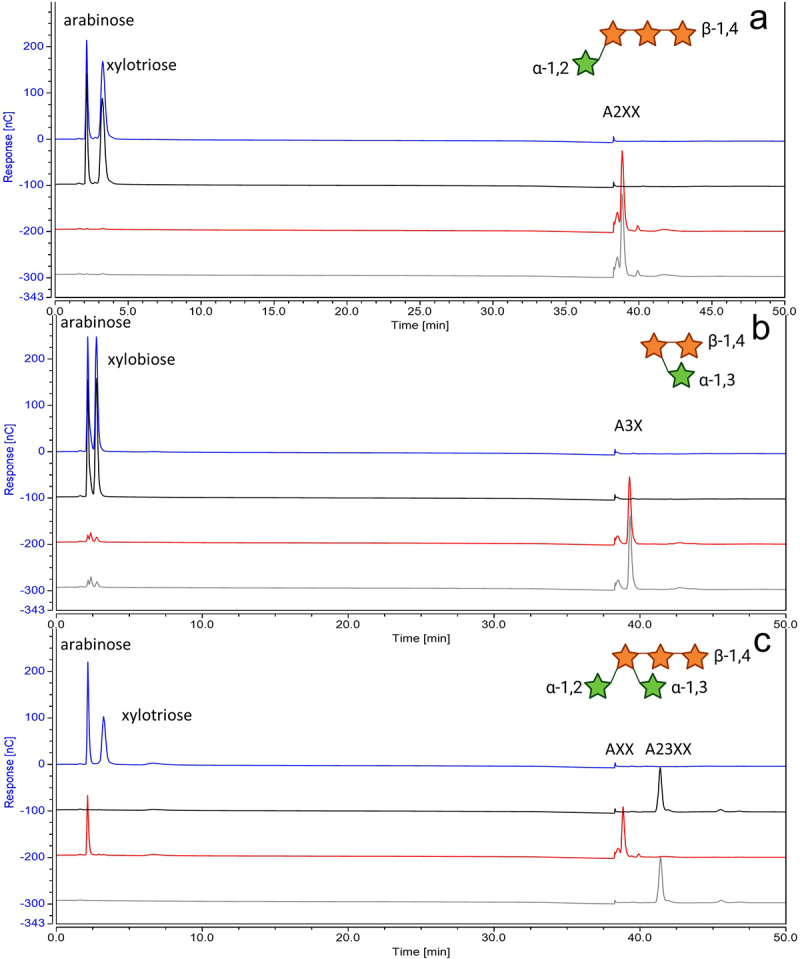
HPAEC-PAD analysis of products from enzymatic reactions of synthetic AX with and without incubation with AxuA_His_/AxuB_His_ see SM6; from the bottom to top: substrate only (gray), with AxuBHis (red), with AxuAHis (black), with both (blue); utilizing A2XX (panel A), A3X (panel B) and A23XX (panel C); the structures of tested synthetic AX are shown in the top-right hand corner. Green Stars indicated arabinose residues and orange stars correspond to xylose units. (structures created with BioRender.com).

AxuA_His_ and AxuB_His_ were further analyzed utilizing various arabinoxylan oligosaccharides (AXOS) and branched arabino oligosaccharides (AOS) with known arabinose linkages as substrates to identify the substrate specificity of each enzyme. AxuB_His_ was shown to exclusively exert activity toward double-branched AXOS: A23XX (results shown in Table 1), while it was shown to be unable to hydrolyze similar double-branched α-1,2 or α-1,3 substitutions from AOS. AxuA_His_ was shown to exhibit α-arabinofuranosidase activity against single α-1,2 or α-1,3 substituted AXOS, removing arabinose moieties from A2XX and XA3XX, while unable to do so for similar arabinose substitutions in AOS. Incubation of A23XX with both AxuA_His_ and AxuB_His_ shows that AxuB_His_ is able to hydrolyze the released product of AxuA_His_ (Figure 6C).

### Structural modelling

In order to understand the different hydrolytic specificities observed for AxuA and AxuB, we predicted structural models for both enzymes using alpha-fold (Fig. S2). Both enzymes possess the typical 5-fold beta-propeller, being a characteristic for members of the GH43 family.^55^ In both enzymes, the catalytic triad (acid, base and pKa modulator) is fully conserved as is true for other arabinofuranosidases described in the cazy database.^17^ In AxuA, E182 may act as catalytic proton donor (acid), D9 may act as catalytic proton acceptor (base) and D129 is proposed to act as pKa modulator (Fig. S2A). In the case of AxuB, D231, D377 and E433 act as base, pKa modulator and acid, respectively (Fig. S2B). However, we also noticed that AxuB has a flexible loop in the active site (residues N274-Q278) that can be the specific substrate determinant impeding access to the active site by certain substrates. In fact, as we describe in Table 1, AxuB can only accommodate A23XX in the active site, which may be explained by the position of this loop. AxuA lacks this loop thus possessing a wider active site enabling the accommodation of other bulkier substrates, such as A3X and A2XX.

### Mutation, complementation, and overexpression

To conclusively demonstrate the importance of AxuA and AxuB in supporting growth of *B. longum* subsp. *longum* NCIMB 8809 on AXR/AXW as the sole carbon and energy source we constructed two insertion mutations in each of the genes in the *axu* gene cluster. Disruption of *axuB* was shown to cause the resulting mutant strain (designated here as *B. longum* subsp. *longum* NCIMB 8809-ΔAxuB) to exhibit significant reduction in growth (*p* ≤ 0.001; compared to the NCIMB 8809 wild type strain) in a medium containing either AXR or AXW, where the observed growth reduction is more pronounced when the mutant strain is grown on AXW. This observed difference may be the result of different levels of arabinose mono-/di-substitutions in AXR and AXW. In contrast, an *axuA* mutation (to generate *B. longum* subsp. *longum* strain NCIMB 8809-ΔAxuA) was shown to result in the complete loss of growth in a medium containing either AXR or AXW, thereby demonstrating that both genes of the *axu* cluster play important roles in facilitating AX metabolism by *B. longum* subsp. *longum* NCIMB 8809.

**Figure 7.**
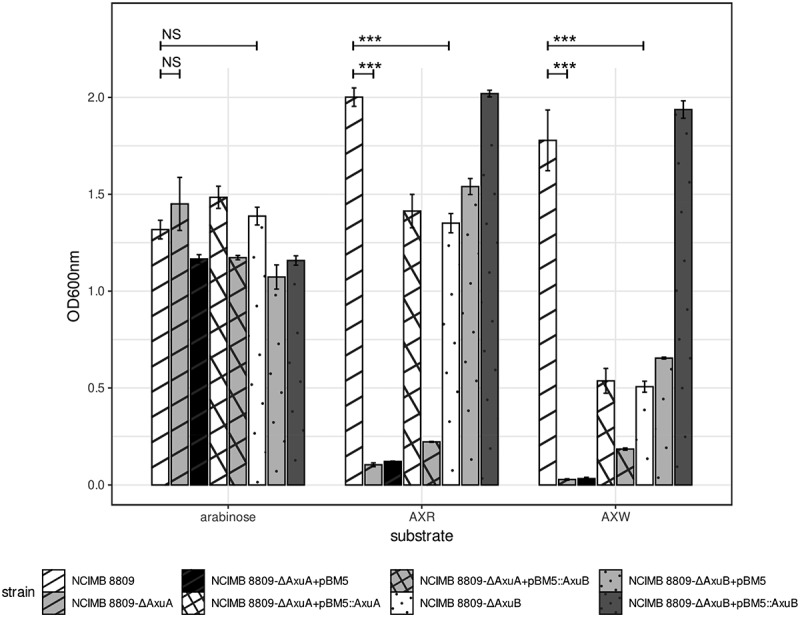
Growth profile of *B. longum* subsp. *longum* NCIMB 8809 mutant and transformants with genes from the *axu* gene cluster OD_600 nm_ after 24 hours anaerobically grown in mMRS + 0.5% (w/v) arabinose, AXR, AXW, and lactose. Asterisks represent a significant difference (*p* ≤ 0.001***) and NS indicates no significant difference (*p* ≥ 0.05).

In order to validate the individual roles of the *axuA* and *axuB* genes in AX metabolism, we cloned each of these two genes (with either the native promoter in the case of *axuA* or a synthetic promoter for *axuB*) into the shuttle vector pBM5 to generate plasmids pBM5:AxuA and pBM5:AxuB, respectively, and used these recombinant plasmids for phenotypic complementation tests. When we introduced plasmid pBM5:AxuA into *B. longum* subsp. *longum* NCIMB 8809-ΔAxuA it resulted in a partially restored ability to utilize AX at a level observed for the NCIMB 8809-ΔAxuB, OD_600 nm_ of over 0.4 on AXW and over 1 on AXR (Figure 7). This indicates that AxuA is produced from pBM5:AxuA, but that its inability to fully restore growth is because the *axuA* mutation in *B. longum* subsp. *longum* NCIMB 8809-ΔAxuA also interrupts transcription of the downstream-located *axuB* (as *axuA* and *axuB* apparently form a single transcriptional unit). The introduction of plasmid pBM5:AxuB into *B. longum* subsp. *longum* NCIMB 8809-ΔAxuB, was shown to fully restore growth of the resulting strain on AXR/AXW to a level that was similar to that of the wild type strain (Figure 7).

**Figure 8.**
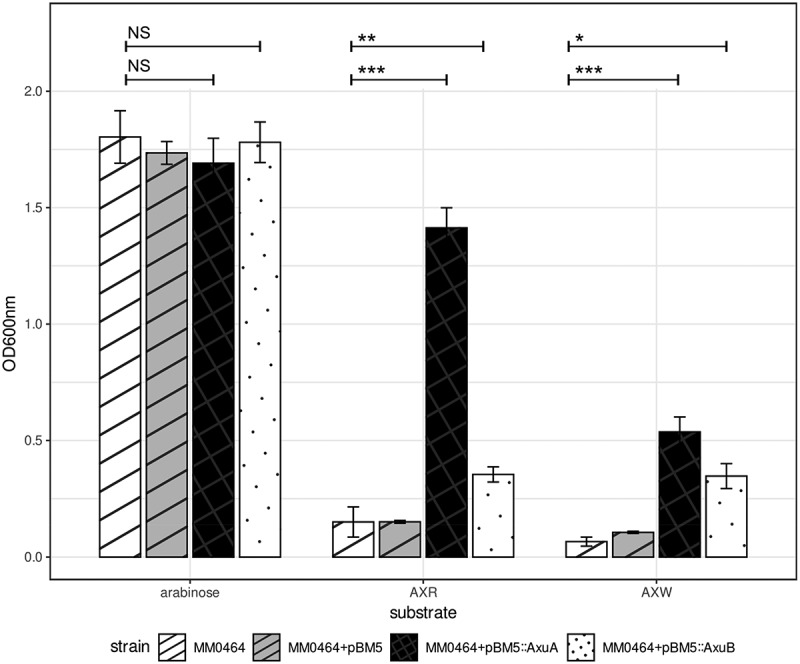
Growth profile of *B. longum* subsp. *longum* MM0464 transformants with genes from the *axu* gene cluster OD_600 nm_ after 24 hours anaerobically grown in mMRS + 0.5% (w/v) arabinose, AXR, AXW, and lactose. Asterisks represent a significant difference (*p* ≤ 0.001***, *p* ≤ 0.01**, *p* ≤ 0.05*) and NS indicates no significant difference (*p* ≥ 0.05).

The importance of AxuA and AxuB for growth on AX by *B. longum* subsp. *longum* strains was further corroborated by introducing pBM5:AxuA or pBM5:AxuB into *B. longum* subsp. *longum* MM0464, a strain which by itself is unable to utilize AX. Introduction of the plasmid-borne *axuA* gene in this strain allowed the resulting transformant to grow on AX, resulting in an OD_600 nm_ of over 0.5 on AXW and over 1 on AXR, while introduction of the plasmid-borne *axuB* gene into the *B. longum* subsp. *longum* MM0464 also resulted in a significant increase (*p* ≤ 0.01 in AXW and *p* ≤ 0.001 in AXR), however, it still does not grow to an OD_600 nm_ above 0.4 (Figure 8). This indicates that both *axuA* and *axuB* are needed to allow vigorous growth on AXR/AXW; several attempts to construct a plasmid containing the complete *axu* cluster failed, probably because of its large size (6 kb).

## Discussion

It has been shown that dietary modulation and an increased plant glycan consumption can influence the microbiome,^56–58^ Specifically, arabinoxylan (AX) and arabinoxylan oligosaccharides (AXOS) are promising prebiotics that have been shown to increase the abundance of members of the genus *Bifidobacterium*, and in particular *B. longum*,^59–61^ Plant/cereal-derived AX may possess a complex structure depending on the source and can in addition to arabinose substitutions carry additional decorations such as glucuronic acids, D-galactose, uronic acids or ferulic acids.^11,62^ The *B. longum* subsp. *longum* taxon is known to be able to metabolize plant-derived poly- and oligosaccharides found in the cell wall of vegetables, fruit, and (cereal) grains, all of which are common components of the adult diet.^11,63^ Various *B. longum* strains, belonging to either subspecies *infantis* or *longum*, are commercially available as probiotics, due to their purported positive effects on human health.^10,64^ Furthermore, stable engraftment of a *B. longum* strain was shown to occur in a sizable proportion of healthy individuals, apparently in part facilitated by glycan-degrading abilities of the strain, thus indicating that diet plays an important role in (long-term) colonization.^65^ Notably, not all *B. longum* subsp. *longum* are able to utilize cereal AX, such as that obtained from wheat or rye, as the sole carbohydrate source, therefore an increase in AX in the diet will not affect the abundance of all *B. longum* subsp. *longum* strains present in the gut. We have shown here that this is correlated to the presence or absence of the *axu* gene cluster in the genome of the strain as its absence renders a strain unable to utilize AX.

Both AxuA and AxuB are produced as extracellular α-arabinofuranosidases, which based on their predicted sortase-motif are presumed to be covalently attached to the cell wall, and which remove arabinose moieties from AX. AxuB targets doubly substituted arabinose-side chains of AX, cleaving one of these linkages thereby releasing monomeric L-arabinose. Based on similarities with AXHd3,^48,66^ we propose that AxuB cleaves the α-1,3 bond, although methylation analysis would be required to validate this notion. Therefore, it is likely that it only needs the double side chains for substrate binding, after which it removes one arabinose moiety from that substrate, thus leaving an AX molecule with just single arabinose substitutions. The latter single substitutions can be removed by AxuA, which is able to hydrolyze singly substituted, α-1,2 and α-1,3 linked arabinose from AX. The requirement of a double arabinose substitution of a xylan substrate in order to be recognized by AxuB may also be the reason why the enzyme failed to exhibit activity toward any of the tested pNp-substrates.

The importance of the *axu* gene cluster in AX metabolism was asserted and investigated in three different ways, gene-trait matching, gene mutation/complementation and gene introduction into *axu*-negative stains. It was observed that only strains including this gene cluster are able to grow on AX. Furthermore, mutations in this gene cluster result in reduced or no growth of *B. longum* subsp. *longum* NCIMB 8809 on cereal-derived AX. Structural variations of AX depending on the source of purification, can also be observed and affects their growth abilities. A mutation in *axuB* results in a more pronounced negative impact on growth in AXW when compared to that in AXR, which indicates that AXW, while having the same arabinose/xylose ratio as AXR (as stated by the supplier), has a higher amount of double arabinose substitutions compared to AXR, on which *B. longum* subsp. *longum* NCIMB 8809 is still able to cleave off single substitutions using AxuA. A mutation in *axuA* results in the complete loss of growth on AXW or AXR. We suspect that the *axuA* mutation we introduced in *B. longum* subsp. *longum* NCIMB 8809 has a polar effect on *axuB* transcription, resulting in the lack of expression of both *axuA* or *axuB*. This also explains why introduction of a plasmid-borne copy of *axuA* did not fully complement the AX growth deficiency of the *axuA* mutant.

Interestingly, introduction of plasmid-borne copies of either *axuA* or *axuB* into *B. longum* subsp. *longum* MM0464, which lacks the *axu* cluster and can thus not grow on AX, allows for the resulting recombinant strains to grow, at least to some degree in AXR and AXW. This means that strain MM0464 must possess a functional arabinose uptake system and suggests that strains, such as MM0464, lacking the *axu gene* cluster are able to cross-feed on arabinose released by *B. longum* subsp. *longum* strains with a functional *axu* cluster.

Variations in AX structure may be the reason for the presence of multiple GH43 enzymes with apparently similar activities, including the previously reported *in vitro* activity of BlArafE and BlArafD on AX.^22^ Our findings clearly show that AxuA and AxuB are required for breakdown of simple AX. Nonetheless, enzymes such as BlArafE and BlArafD or those encoded by the *aau* gene cluster may be required for hydrolysis of other arabinose-containing substitutions present in more complex AX, such as that from corn.

**Figure 9.**
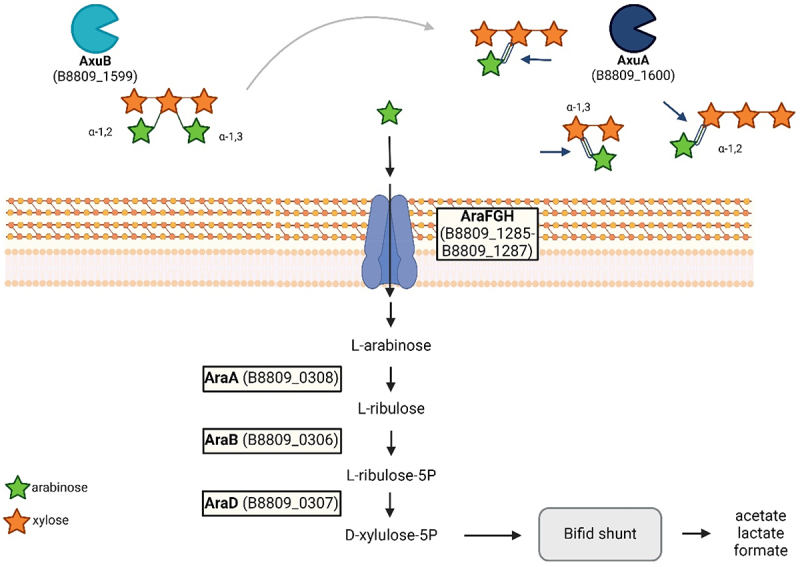
Model of AX degradation of AxuA and AxuB and arabinose metabolism in *B. longum* susp. *longum* locus tags for genes in *B. longum* susp. longum NCIMB8809, see text for further details. Created with BioRender.com.

Our findings have shown that AxuA and AxuB are both required for the efficient utilization of cereal-derived AX by *B. longum* subsp. *longum* NCIMB 8809. Our transcriptome analysis also identified candidate genes for the other required parts of this AX-utilization pathway (schematically outlined in Figure 9). Extracellularly released arabinose is assumed to be imported into the cell by an ABC-type transporter (represented by AraFGH and encoded in *B. longum* subsp. *longum* NCIMB 8809 by genes with locus tags B8809_1285–1287), where it then undergoes a multiple step conversion into D-xylulose 5-phosphate (performed by AraABD and encoded in *B. longum* subsp. *longum* NCIMB 8809 by genes with locus tags B8809_306–308). In the latter predicted pathway AraA (a putative L-arabinose isomerase) converts arabinose into L-ribulose, AraB (ribulose kinase) converts it into ribose 5-phosphate before AraD (predicted Ribulose-5P-epimerase converts it into D-xylulose 5-P.^23^ This metabolite is then introduced in the pentose sugar branch of the fructose-6-phosphoketolase pathway or ‘Bifid’ shunt.^45,67^

In conclusion, this study has identified the key enzymes and associated metabolic pathway encoded by certain *B. longum* subsp. *longum* strains to allow fermentative metabolism of wheat- and rye-derived AX.

## Supplementary Material

Supplemental Material

## Data Availability

The sequencing data that support the findings of this study are openly available in NCBI at https://www.ncbi.nlm.nih.gov/ within the BioProject PRJNA1073879 and PRJNA1082215.
