# Supplementary material for: Two extracellular α-arabinofuranosidases are required for cereal-derived arabinoxylan metabolism by Bifidobacterium longum subsp. longum
Source: Gut Microbes. 2024 May 16;16(1):2353229. doi: 10.1080/19490976.2024.2353229 (PMC11318964; doi:10.1080/19490976.2024.2353229)
Supplement: Supplemental Material [file KGMI_A_2353229_SM2737.docx]

# **Supplementary methods:**

## Supplementary Method S1: *In silico* design and construction of pFREM2 vector

Restriction-Modification motifs for *B. longum* NCIMB 8809 and CCUG30698 strains had previously been identified^1^ using a combination of SMRT/bisulfite sequencing and comparative genome analysis. The pFREM2 vector was constructed using the pFREM28 suicide vector described previously described^2^ as a template sequence and removing in silico all the Restriction-Modification target sites assiciated with the B. longum strains. All sequence editing and removal of restriction enzyme recognition sites were performed manually using the SnapGene v2.32 and Artemis^3^ software tools. Finally, BLASTP alignment was used to ensure the preservation of the sequence identity of the Em^R^ antibiotic selection marker^4^ after the introduced base substitutions. The sequence of the resulting *in silico* constructed vector, which was designated pFREM2, was synthesized by BaseClear (Leiden, The Netherlands), and made available by this provider as a cloned and sequence verified fragment in *E. coli* vector pUC57. Unique restriction sites (XbaI) had been included at the left and right end of pFREM2 to allow for excision from pUC57 by restriction digestion. Following self-ligation and circularization of the obtained pFREM2 vector, the conditional replication functionality was confirmed using *E. coli* strain EC101.

## Supplementary Method S2: Sequencing of *B. longum* subsp. *longum* strains

The 25 *B. longum* subsp. *longum* strains isolated as part of the Microbe Mom study^5^ were send to Plasmidsaurus (Eugene, OR, USA) for hybrid Oxford Nanopore Technologies and Illumina sequencing. For MM0003, MM0024, MM0160, MB0212, MB0308 and MM0464 cultures were used for genomic DNA extraction using a nucleobond genomic DNA isolation kit with AXG-100 columns (Macherey-Nagel, Düren, Germany). For the 19 remaining strains, cells were harvested from 10 ml culture with an OD_600nm_ of approximately 1, washed in phosphate-buffered saline (Sigma-Aldrich, Steinheim, Germany) and resuspended in 500μL DNA/RNA shields (Zymo Research, Orange, CA, USA) and send to Plasmidsaurus for gDNA extraction and sequencing. The reads were assembled by Plasmidsaurus, using Filtlong v0.2.1 (https://github.com/rrwick/Filtlong.), Miniasm v0.3^6^, Flye v2.9.1^7^, Medaka v1.8.0 (https://github.com/nanoporetech/medaka), Bakta v1.6.1^8^, Bandage v0.8.1^9^, CheckM v1.2.2^10^, Mash v2.3^11^ against RefSeq genomes and plasmids, Sourmash v4.6.1^12^ against GenBank and polished using Polypolish v0.5.0^13^, resulting in a full-length contig for each of the genomes.

## Supplementary Method S3: Cloning of *blArafB* and *blArafC* into *B. longum* subsp. *longum* NCIMB 8809

*blArafB* (BLLJ_1853) and *blArafC* (BLLJ_1852) were cloned into the *B. longum* subsp. *longum* NCIMB 8809, by amplifying the genes, individually and together, by PCR (Q5 High-Fidelity DNA polymerase; NEB; primers can be found in Tab. S2), using genomic DNA extracted from *B. longum* subsp. *longum* JCM 1217 as a template and cloned into pBM5 (see method S5), using ApaI and NheI as restriction enzyme sites and DH5α as cloning host, before transferring the individual recombinant plasmids into EC101 cells harbouring pNZEM. To validate their genetic integrity all constructs were verified by DNA sequencing (Plasmidsaurus, Eugene, OR, USA). The plasmids were extracted and transformed into *B. longum* subsp. *longum* NCIMB 8809 as described above.

## Supplementary Method S4: Construction of pBM5

pBM5 was generated by PCR amplification (Q5 High-Fidelity DNA polymerase, NEB, Ibswick, MA, US) of the ColEI origin, pBC1.2 backbone and *tetM*, using pAM5 as template, removing its Amp^R^ site and including a promoter for *tetM* into the primer. The PCR product was ligated to itself, using MfeI restriction sites, and introduced into *E. coli* DH5α as a cloning host^14^.

## Supplementary Method S5: *In silico* analysis

Artemis genome browser^3^ was used to visualise genetic features using the annotated genome of *B. longum* subsp. *longum* NCIMB 8809^1^. Protein function and homology searches were performed using BlastP, HHPred and HMMER^15–17^. SignalP was used to predict if a given protein sequence is secreted or cytoplasmic ^18^ and TMHMM 2.0 was used to predict membrane spanning domains^19^. CAZy was used to determine the determine the subfamily classification of the GH43 family and further confirmed that both enzymes belong to the corresponding subfamilies^20^.

## Supplementary Method S6: HPAEC-PAD analysis of A2XX, A3X and A23XX

Enzymatic assays were performed for substrates A2XX, A3X and A23XX, which were each incubated for 30 min with AxuB_His_ or AxuA_His_, or a combination of these two enzymes under the previously described conditions and then analysed by High-Performance Anion Exchange Chromatography–Pulsed Amperometric Detection (HPAEC-PAD). A gradient of 100 mM KOH from 0 to 50 min together with methanesulfonic acid (KMSA): 0 mM from 0 to 15 min, 10 mM to 100 mM from 15 to 35 min, 10 mM from 35 to 45 min, 0 mM from 45 to 50 min was used.

## Supplementary Method S7: Structural prediction with AlphaFold

3-D structures of AxuA (B8809_1599) and AxuB (B8809_1600) were modelled using AlphaFold2 colab software (https://colab.research.google.com/github/sokrypton/ColabFold/blob/main/AlphaFold2.ipynb#scrollTo=UGUBLzB3C6WN). To model arabinose in the active site of both AxuA and AxuB, we used the GH43 arabinofuranosidase enzyme from Humicola insolens (PDB 3zxk) as template. Structures were visualised using Pymol (The PyMOL Molecular Graphic system, version 2.0 Schrodinger, LLC).

# **Supplementary tables:**

## Supplementary Table S1: Bacterial strains and plasmids used in this study.

| Strain | Features | Refences/source |
| --- | --- | --- |
| *B. longum* subsp*. longum* |  |  |
| NCIMB 8809 | isolated from baby stool | NCIMB |
| JCM 1217 | Isolated from adult stool | JCM |
| MB0044 | isolated from baby stool | ^5^ |
| MB0212 | isolated from baby stool | ^5^ |
| MB0308 | isolated from baby stool | ^5^ |
| MB0318 | isolated from baby stool | ^5^ |
| MM0003 | isolated from adult stool | ^5^ |
| MM0024 | isolated from adult stool | ^5^ |
| MM0160 | isolated from adult stool | ^5^ |
| MM0259 | isolated from adult stool | ^5^ |
| MM0286 | isolated from adult stool | ^5^ |
| MM0289 | isolated from adult stool | ^5^ |
| MM0302 | isolated from adult stool | ^5^ |
| MM0307 | isolated from adult stool | ^5^ |
| MM0321 | isolated from adult stool | ^5^ |
| MM0360 | isolated from adult stool | ^5^ |
| MM0362 | isolated from adult stool | ^5^ |
| MM0364 | isolated from adult stool | ^5^ |
| MM0369 | isolated from adult stool | ^5^ |
| MM0375 | isolated from adult stool | ^5^ |
| MM0380 | isolated from adult stool | ^5^ |
| MM0441 | isolated from adult stool | ^5^ |
| MM0450 | isolated from adult stool | ^5^ |
| MM0464 | isolated from adult stool | ^5^ |
| MM0465 | isolated from adult stool | ^5^ |
| MM0492 | isolated from adult stool | ^5^ |
| MM0494 | isolated from adult stool | ^5^ |
| NCIMB 8809- Δ AxuB | NCIMB 8809 with an insertion mutation in *axuB* | this work |
| NCIMB 8809- Δ AxuB-pBM5 | NCIMB 8809- Δ AxuB containing pBM5 | this work |
| NCIMB 8809- Δ AxuB-pBM5::AxuB | NCIMB 8809- Δ AxuB containing pBM5::*axuB* | this work |
| NCIMB 8809- Δ AxuA | NCIMB 8809 with an insertion mutation in *arfB* | this work |
| NCIMB 8809- Δ AxuA-pBM5 | NCIMB 8809- Δ AxuA containing pBM5 | this work |
| NCIMB 8809- Δ AxuA-pBM5::AxuB | NCIMB 8809- Δ AxuA containing pBM5::*axuB* | this work |
| NCIMB 8809- Δ AxuA-pBM5::AxuA | NCIMB 8809-A Δ xuA containing pBM5::*arfB* | this work |
| NCIMB 8809- Δ AxuA-pBM5::AxuB+AxuA | NCIMB 8809- Δ AxuB containing pBM5::*axuB+arfB* | this work |
| NCIMB 8809-pBM5 | NCIMB 8809 containing pBM5 | this work |
| NCIMB 8809-pBM5::BlArafB | NCIMB 8809 containing pBM5::*blArafB* | this work |
| NCIMB 8809-pBM5::BlArafC | NCIMB 8809 containing pBM5*::blArafC* | this work |
| *E.coli* |  |  |
| XL1 Blue | cloning host , recA1, Tet^r^ | Stratagene |
| EC101 | cloning host, repA^+^ km^r^ | ^21^ |
| DH5α | cloning host, recA1 | Invitrogen |
| BL21 | cloning host, T7^-^ | Stratagene |
| 10-beta | cloning host | New England Biolabs |
| Plasmids |  |  |
| pET28b | *E.coli* expression vector with N-terminal His tag | Novagen |
| pET28b::AxuB | pET28b harbouring *axuB* | this work |
| pET28b::AxuA | pET28b harbouring *arfB* | this work |
| pFREM2 | pFREM28 - R-M motif free for *B. longum* | this work |
| pFREM2::AxuB | pFREM2 harbouring an internal fragment of *axuB* | this work |
| pFREM2::AxuA | pFREM2 harbouring an internal fragment of *arfB* | this work |
| pAM5 | Shuttle vector based on pBC1 & pUC19-Tc^r^-Amp^R^ | ^22^ |
| pBM5 | pBC1 -Tc^r^ | this work |
| pBM5::AxuB | pBM5 harbouring *axuB* | this work |
| pBM5::AxuA | pBM5 harbouring *arfB* | this work |
| pBM5::AxuB+AxuA | pBM5 harbouring *axuB + arfB* | this work |
| pBM5::BlArafB | pBM5 harbouring *blArafB* | this work |
| pBM5::BlArafC | pBM5 harbouring *blArafC* | this work |
| pNZEM | Gene expression vector, Em_r_ | ^2^ |

**Supplementary Table S2: Oligonucleotides used in this study.**

| **Target** | **Primer** | **Sequence (5’ to 3’)** | **GC%** | **Length (bp)** | **Size (bp)** | **T_m_ (℃)** |
| --- | --- | --- | --- | --- | --- | --- |
| B8809_1599 | ArfB_His_F | atgttgaacacggatgttcc | 45 | 20 | 2619 | 63 |
|  | ArfB_His_R | accagttgatgacagttcag | 45 | 20 |  |  |
|  | ArfB_IM_F | gttattcgtgtgtcgaaggacg | 50 | 22 | 491 | 66 |
|  | ArfB_IM_R | tcatcagtgaactgttcggc | 50 | 20 |  |  |
|  | ArfB_C_F | ggttaaaagacttatgaattatttacgac | 28 | 29 | 2897 | 61 |
|  | ArfB_CR | cgatatttcagcgcgaagag | 50 | 20 |  |  |
| B8809_1600 | ArfA_His_F | gaacccgtcgaattggtcg | 58 | 19 | 2334 | 67 |
|  | ArfA_His_R | accggtttgtgccaaatccac | 52 | 21 |  |  |
|  | ArfA_IM_F | gaaaaccggtcgtgtggttg | 55 | 20 | 468 | 68 |
|  | ArfA_IM_R | ccactaccacctaacgttccag | 55 | 22 |  |  |
|  | ArfA_C_F | agctttataggcgatgagtgg | 48 | 21 | 2935 | 63 |
|  | ArfA_C_R | cgcattaccaagaggtaactag | 45 | 22 |  |  |
| BLLJ_1852 | *blArafC_F* | gcggaaagaggaaaccaatg | 50 | 20 | 3783 | 65 |
|  | *blArafB_R* | tttcgcgattggaggcttac | 50 | 20 |  |  |
| BLLJ_1853 | *blArafB_F* | gaggagaaacatgggaaagc | 50 | 20 | 3322 | 64 |
|  | *blArafB_R* | gtctacgctgcaaacagttc | 50 | 20 |  |  |

**Supplementary Table S3: Use AOS and AXOS with corresponding abbreviation and structure.** Green stars are indicating arabinose units and orange stars are indicating to xylose units. (Structures created with BioRender.com)

| Name | Abbreviation | Structure |
| --- | --- | --- |
| 3^2^-α-L-arabinofuranosyl-arabinotriose | AA3A | 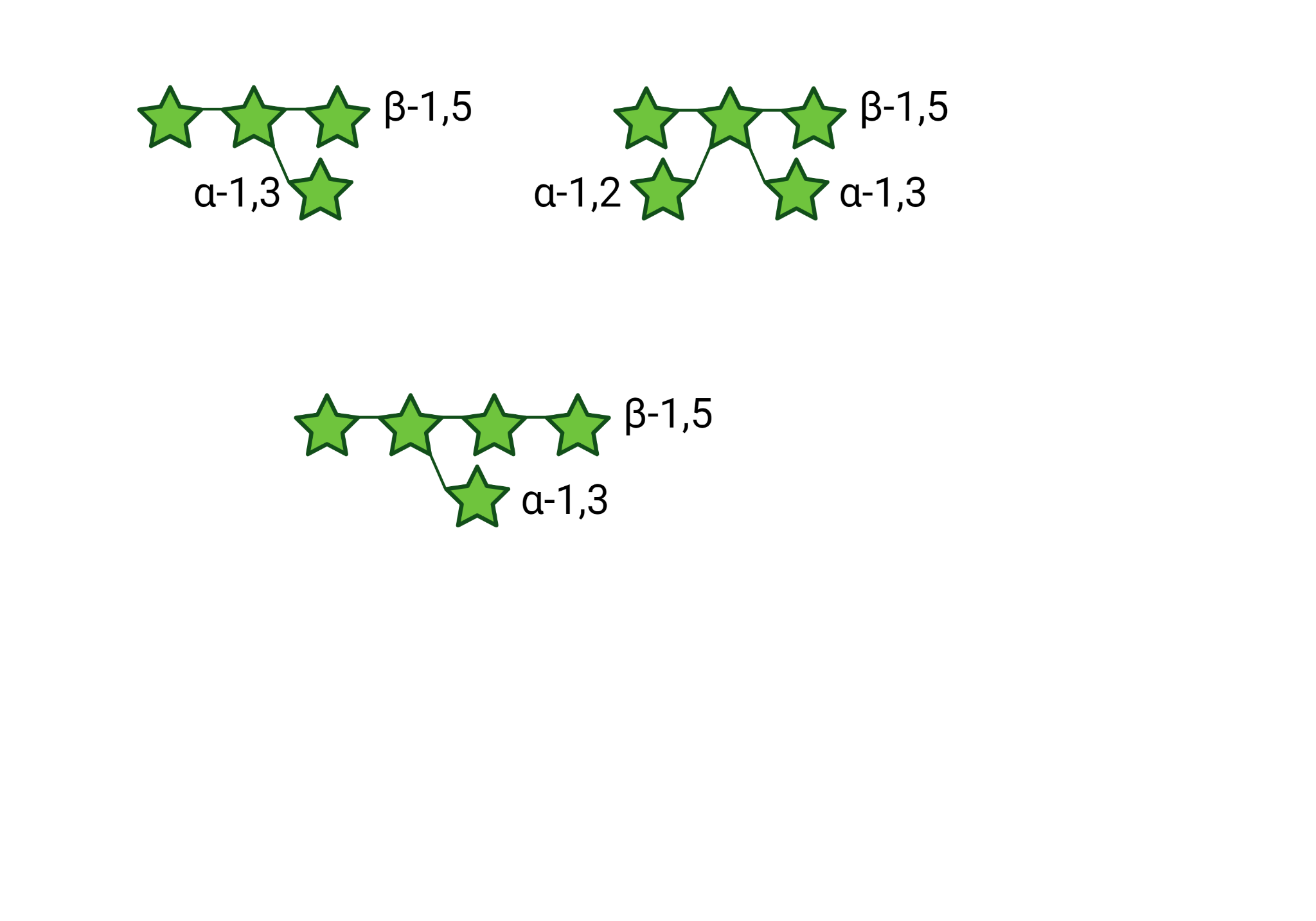 |
| 2^2^,3^2^-di-α-L-arabinofuranosyl-arabinotriose + 3^3^-α-L-arabinofuranosyl-arabinotetraose | AA23A + AA3AA | 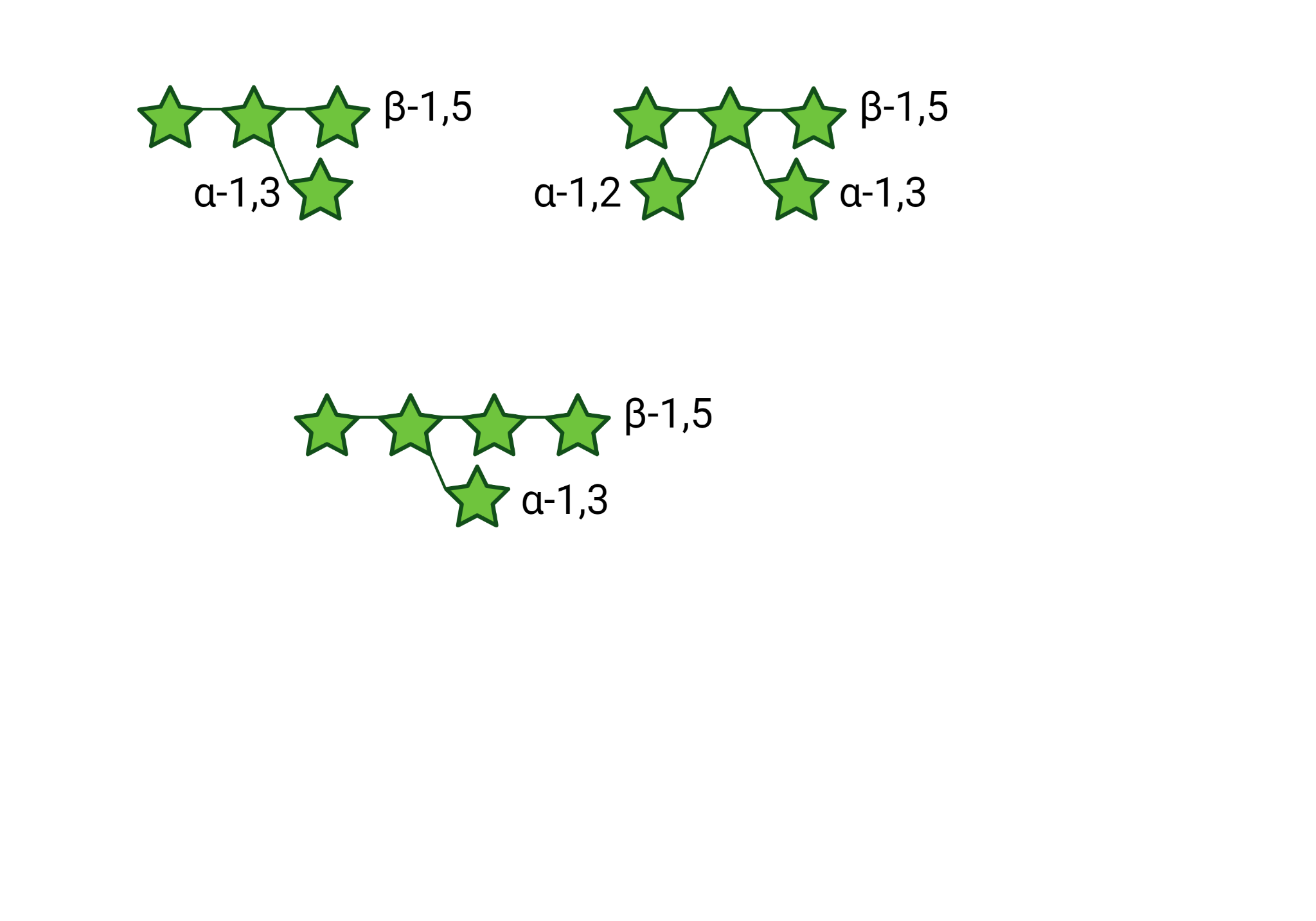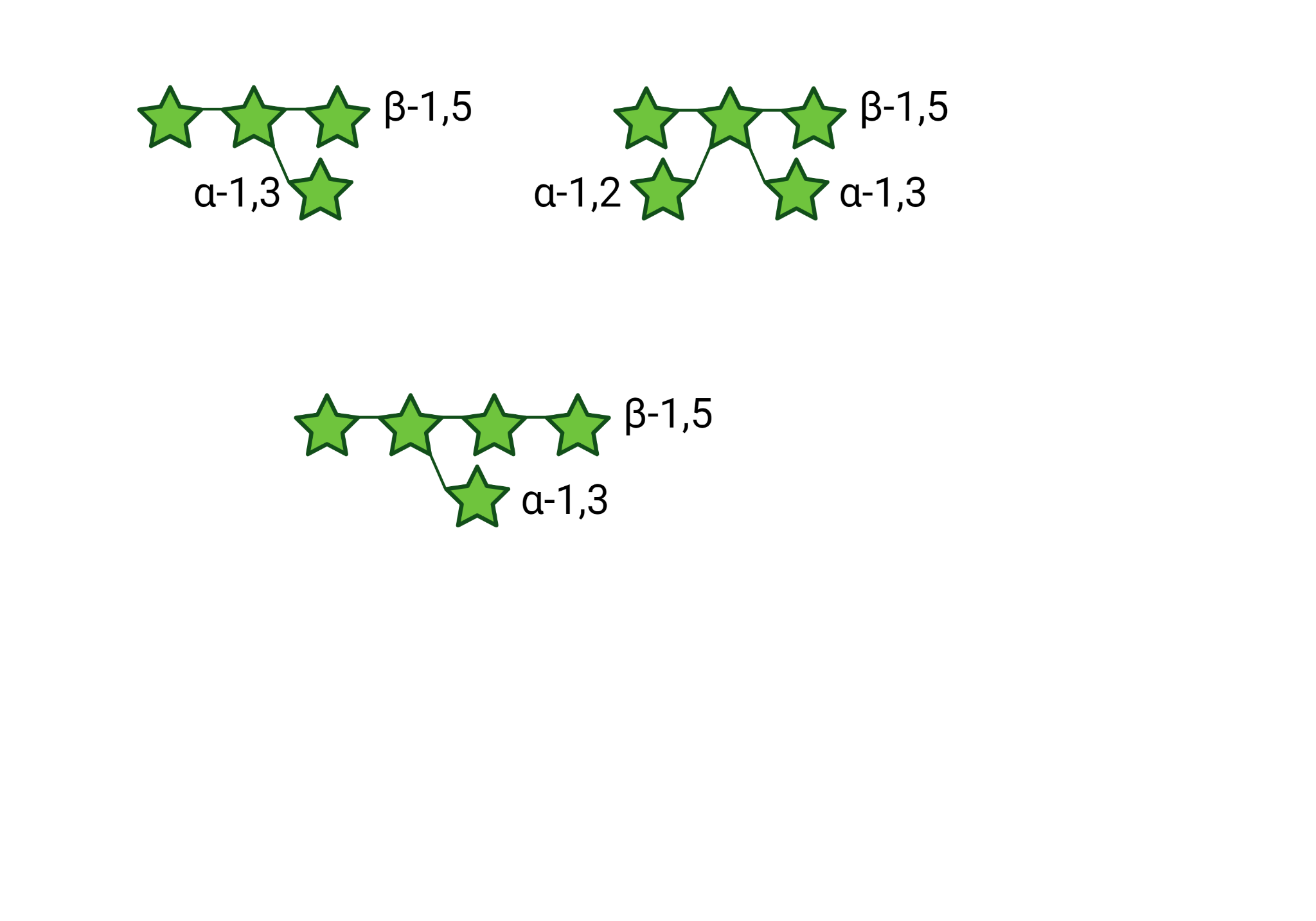 |
| 2^3^-α-L-arabinofuranosyl-xylotriose | A2XX | 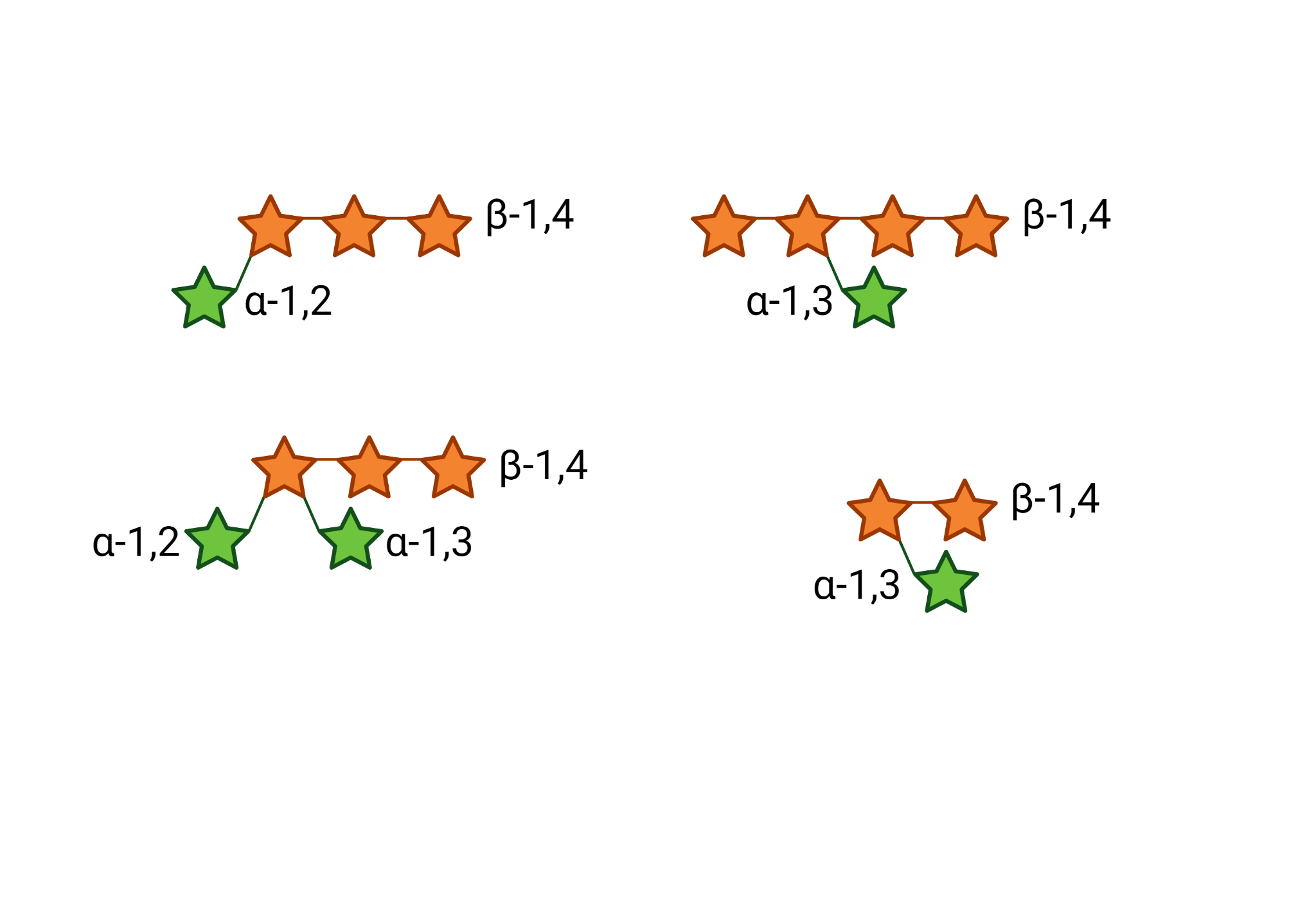 |
| 2^3^,3^3^-di-α-L-arabinofuranosyl-xylotriose | A23XX | 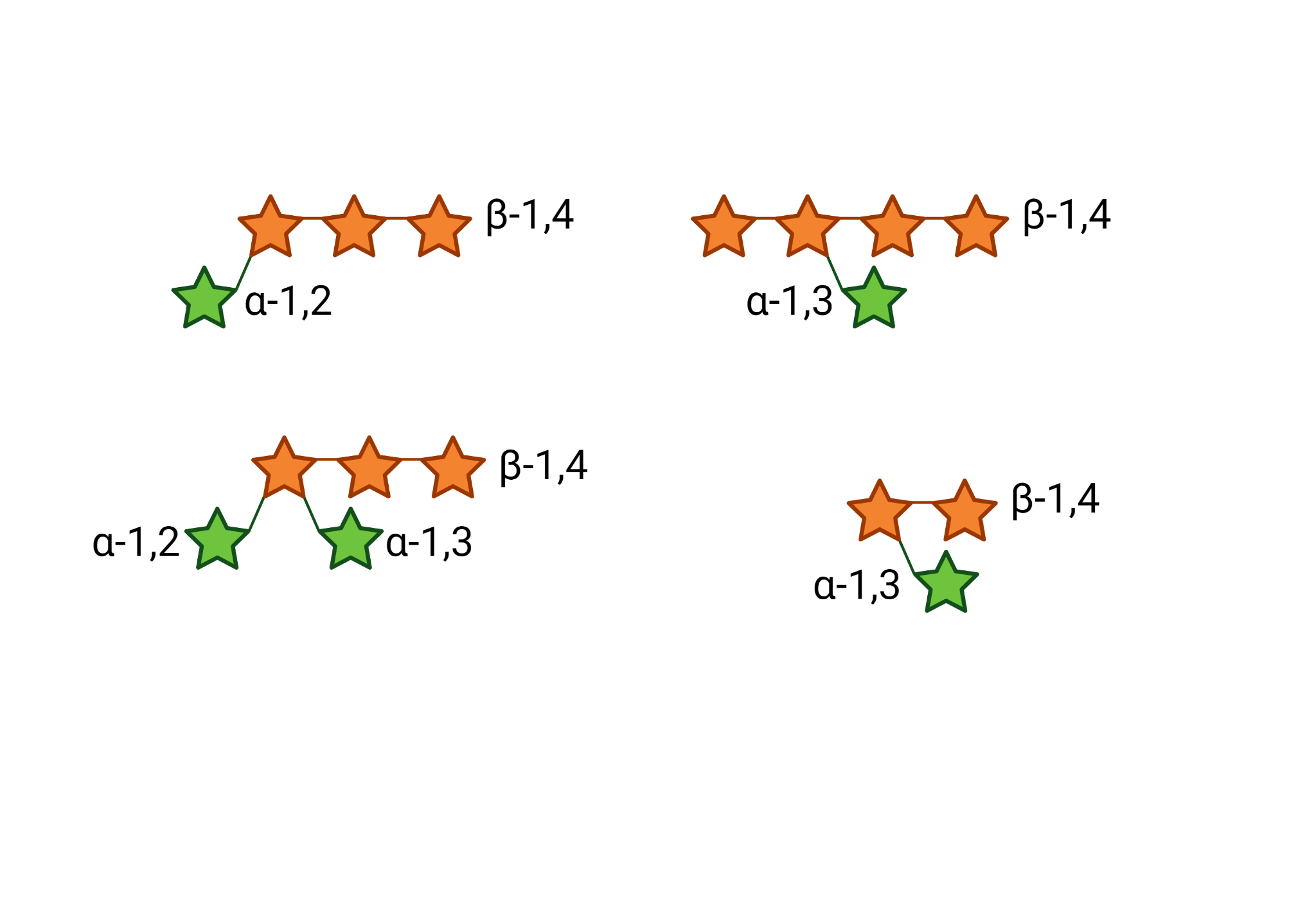 |
| 3^2^-α-L-Arabinofuranosyl-xylobiose | A3X | 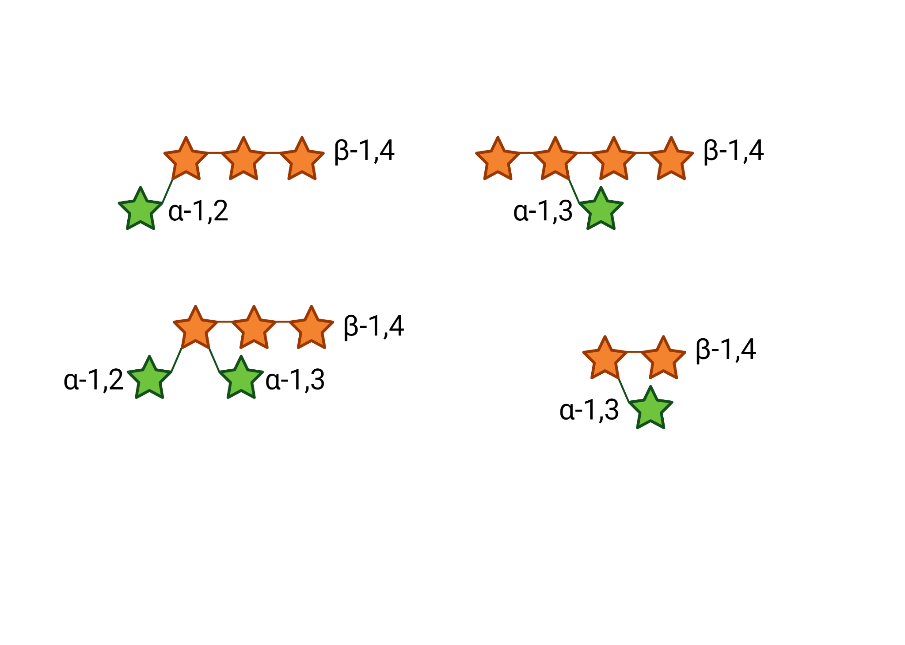 |

## Supplementary Table S4: General features of sequenced *B. longum subsp. longum* strains.

| Strain name | Genome size | Genes annotated | GC content | Sequencing coverage | Plasmid present | GenBank Accession number |
| --- | --- | --- | --- | --- | --- | --- |
| MB0044 | 2.5 Mb | 2,176 | 60.21% | 100% | 0 | CP146844 |
| MB0212 | 2.4 Mb | 1,968 | 59.92% | 100% | 1 | CP146014-CP146015 |
| MB0308 | 2.3 Mb | 2,002 | 60.15% | 100% | 0 | CP146700 |
| MB0318 | 2.4 Mb | 2,064 | 59.93% | 100% | 0 | CP146699 |
| MM0003 | 2.4 Mb | 2,064 | 60.29% | 99.91% | 3 | CP146904-CP146907 |
| MM0024 | 2.4 Mb | 2,066 | 59.78% | 100% | 2 | CP146639-CP146641 |
| MM0160 | 2.5 Mb | 2,097 | 60.02% | 99.77% | 0 | CP146698 |
| MM0259 | 2.5 Mb | 2.146 | 60.27% | 100% | 0 | CP146697 |
| MM0286 | 2.4 Mb | 2,043 | 60.29% | 99.69% | 2 | CP146642-CP146644 |
| MM0289 | 2.5 Mb | 2,162 | 60.17% | 100% | 2 | CP146901-CP146903 |
| MM0302 | 2.2 Mb | 1,931 | 59.97% | 100% | 0 | CP146696 |
| MM0307 | 2.4 Mb | 2,034 | 60.13% | 100% | 0 | CP146695 |
| MM0321 | 2.7 Mb | 2,403 | 59.75% | 100% | 2 | CP146636-CP146638 |
| MM0360 | 2.3 Mb | 1,965 | 60.18% | 100% | 1 | CP146634-CP146635 |
| MM0362 | 2.5 Mb | 2,230 | 60.09% | 100% | 3 | CP146630-CP146633 |
| MM0364 | 2.6 Mb | 2,220 | 60.24% | 100% | 0 | CP146694 |
| MM0369 | 2.4 Mb | 2,145 | 60.28% | 100% | 1 | CP146491-CP146492 |
| MM0375 | 2.4 Mb | 2,061 | 59.77% | 100% | 1 | CP146645-CP146646 |
| MM0380 | 2.3 Mb | 1,984 | 60.02% | 100% | 0 | CP146484 |
| MM0441 | 2.4 Mb | 2,044 | 60.17% | 100% | 1 | CP146496-CP146497 |
| MM0450 | 2.4 Mb | 1,995 | 60.17% | 99.92% | 2 | CP146493-CP146495 |
| MM0464 | 2.2 Mb | 1,931 | 59.97% | 100% | 0 | CP146483 |
| MM0465 | 2.3 Mb | 1,997 | 60.15% | 100% | 0 | CP146485 |
| MM0492 | 2.5 Mb | 2,228 | 59.82% | 99.77% | 1 | CP146645-CP146646 |
| MM0494 | 2.3 Mb | 1,935 | 60.19% | 99.81% | 3 | CP146487-CP146490 |

**Supplementary Table S5: A selection of hits generated when aligning the enzymes with HHPred and HMMER**

| Enzyme | TMHMM | Hit | Methode | Substrate | Organism | Reference |
| --- | --- | --- | --- | --- | --- | --- |
| AxuB | Extracellular - 2 trans- | Glyco_hydro_43 | HMMER | N/A | N/A | ^16^ |
|  | membrane | GH43_C2 | HMMER | N/A | N/A | ^16^ |
|  | domains | Arabinofuranosidase (*HiAXHd3*) | HHpred | AX | *Humicola insolens* | ^23^ |
|  |  | β-xylosidase | HMMER | AX | *B.* *longum* subsp. *longum* NCC2705 | ^24^ |
|  |  | α-arabinofuranosidase (AXHd3) | HHpred | AX | *B.* *adolecentis* DSM 20083 | ^25^ |
| AxuA | Extracellular - 2 trans- | Glyco_hydro_43 | HMMER | N/A | N/A | ^16^ |
|  | membrane domains | endo-1,5-alpha-L-arabinanase (ARN3) | HHpred | AX | *Thermotoga petrophila* | ^26^ |
|  |  | endo-1,5-alpha-L-arabinanase (BsArb43B) | HHpred | AX | *Bacillus subtilis* | ^27^ |
|  |  | endo-1,4-beta-xylanase (BsAXH-m2,3) | HHpred | AX | *B. subtilis* | ^28^ |
|  |  | endoxylanase (XynD) | HMMER | AX | *B.* *longum* subsp. *longum* NCC2705 | ^29^ |
|  |  | endoxylanase (XynD) | HMMER | AX | *Bacillus polymyxa* | ^30^ |

# **Supplementary figures:**

**
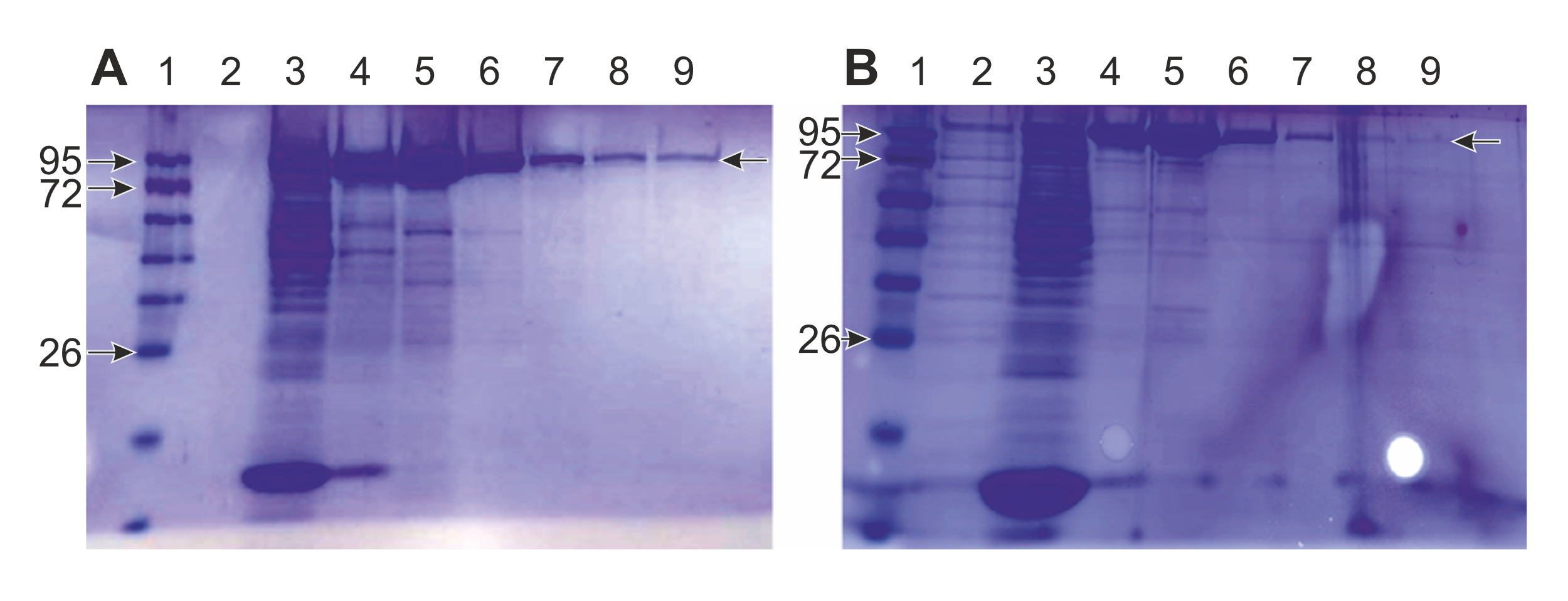
**

**Supplementary Figure S1: SDS-PAGE analysis of AxuA and AxuB**.

**
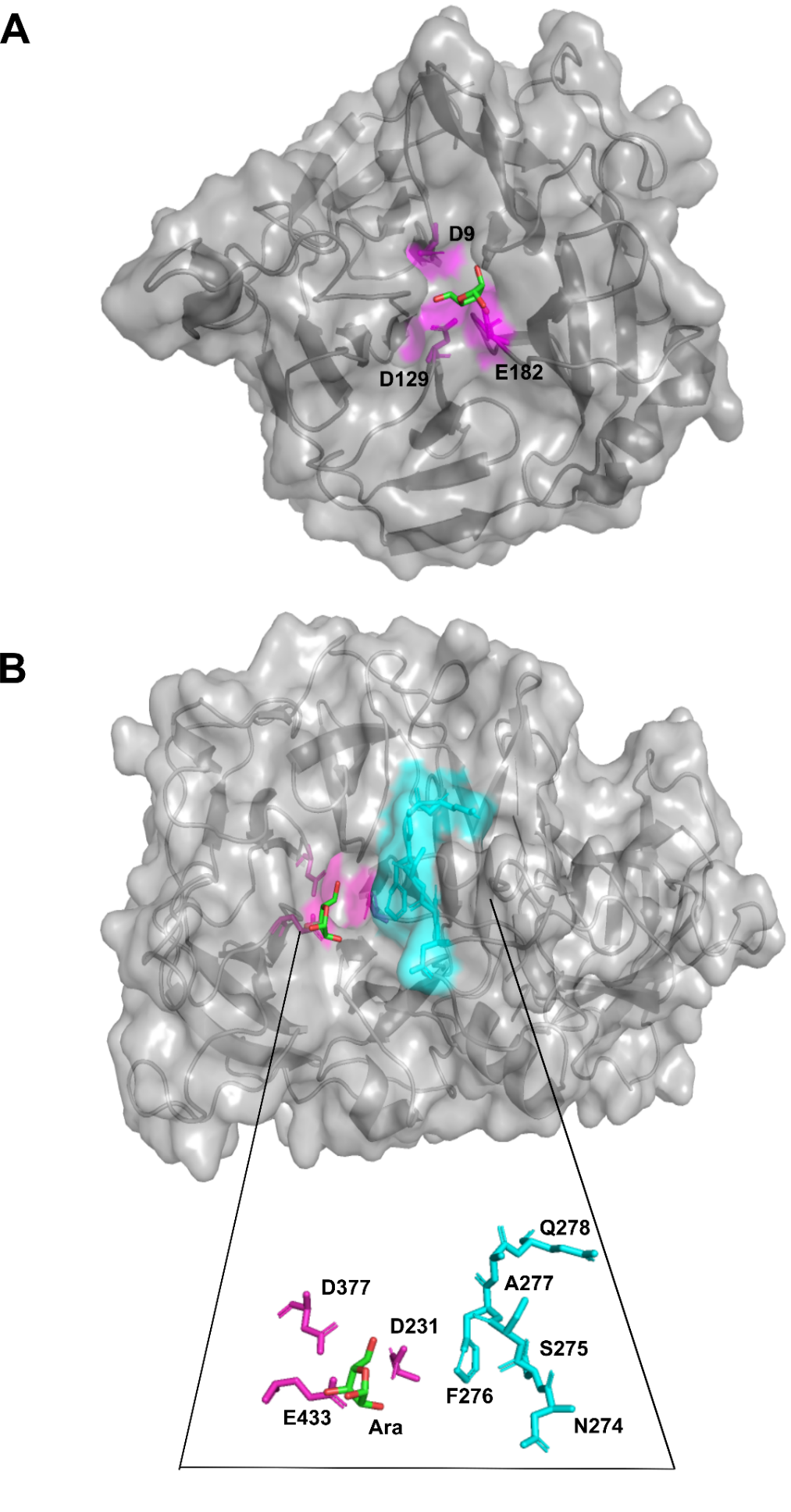
**

**Supplementary Figure S2. Structural models of AxuA (A) and AxuB (B).**

**Supplementary Figure Legends**

**Supplementary Figure S1: SDS-PAGE analysis of AxuA and AxuB**. SDS-PAGE analysis of AxuB_His_ (B8809_1599; A) and AxuA_His_ (B8809_1600; B). SDS-PAGE gels are all loaded in the following order: a protein ladder (lane 1), supernatant (lane 2), column wash (lane 3), elution fraction (lane 4), used elution fraction (lane 5-8). Protein sizes are indicated in kDa; and the arrow indicates the protein of interest.

**Supplementary Figure S2. Structural models of AxuA (B) and AxuB (A).** AxuB has the catalytic acid, base and pKa modulator highlighted in pink. AxuA has also the catalytic acid, base and pKa modulator highlighted in pink but also the critical loop in cyan.

# **Supplementary References:**

1. O’Callaghan A, Bottacini F, O’Connell Motherway M, van Sinderen D. Pangenome analysis of *Bifidobacterium longum* and site-directed mutagenesis through by-pass of restriction-modification systems. *BMC Genomics*. 2015;16(1). doi:10.1186/s12864-015-1968-4

2. Hoedt EC, Bottacini F, Cash N, Bongers RS, van Limpt K, Ben Amor K, Knol J, MacSharry J, van Sinderen D. Broad Purpose Vector for Site-Directed Insertional Mutagenesis in *Bifidobacterium breve*. *Front Microbiol*. 2021;12. doi:10.3389/fmicb.2021.636822

3. Carver T, Harris SR, Berriman M, Parkhill J, McQuillan JA. Artemis: An integrated platform for visualization and analysis of high-throughput sequence-based experimental data. *Bioinformatics*. 2012;28(4):464-469. doi:10.1093/bioinformatics/btr703

4. Bottacini F, Morrissey R, Roberts RJ, James K, Van Breen J, Egan M, Lambert J, Van Limpt K, Knol J, Motherway MOC, et al. Comparative genome and methylome analysis reveals restriction/modification system diversity in the gut commensal Bifidobacterium breve. *Nucleic Acids Res*. 2018;46(4):1860-1877. doi:10.1093/nar/gkx1289

5. Feehily C, O’Neill IJ, Walsh CJ, Moore RL, Killeen SL, Geraghty AA, Lawton EM, Byrne D, Sanchez-Gallardo R, Nori SRC, et al. Detailed mapping of *Bifidobacterium* strain transmission from mother to infant via a dual culture-based and metagenomic approach. *Nat Commun*. 2023;14(1). doi:10.1038/s41467-023-38694-0

6. Li H. Minimap and miniasm: Fast mapping and de novo assembly for noisy long sequences. *Bioinformatics*. 2016;32(14):2103-2110. doi:10.1093/bioinformatics/btw152

7. Kolmogorov M, Yuan J, Lin Y, Pevzner PA. Assembly of long, error-prone reads using repeat graphs. *Nat Biotechnol*. 2019;37(5):540-546. doi:10.1038/s41587-019-0072-8

8. Schwengers O, Jelonek L, Dieckmann MA, Beyvers S, Blom J, Goesmann A. Bakta: Rapid and standardized annotation of bacterial genomes via alignment-free sequence identification. *Microb Genom*. 2021;7(11). doi:10.1099/MGEN.0.000685

9. Wick RR, Schultz MB, Zobel J, Holt KE. Bandage: Interactive visualization of de novo genome assemblies. *Bioinformatics*. 2015;31(20):3350-3352. doi:10.1093/bioinformatics/btv383

10. Parks DH, Imelfort M, Skennerton CT, Hugenholtz P, Tyson GW. CheckM: Assessing the quality of microbial genomes recovered from isolates, single cells, and metagenomes. *Genome Res*. 2015;25(7):1043-1055. doi:10.1101/gr.186072.114

11. Ondov BD, Starrett GJ, Sappington A, Kostic A, Koren S, Buck CB, Phillippy AM. Mash Screen: High-throughput sequence containment estimation for genome discovery. *Genome Biol*. 2019;20(1). doi:10.1186/s13059-019-1841-x

12. Titus Brown C, Irber L. sourmash: a library for MinHash sketching of DNA. *The Journal of Open Source Software*. 2016;1(5):27. doi:10.21105/joss.00027

13. Wick RR, Holt KE. Polypolish: Short-read polishing of long-read bacterial genome assemblies. *PLoS Comput Biol*. 2022;18(1). doi:10.1371/journal.pcbi.1009802

14. Green M, Sambrook J. Transformation of *Escherichia coli* by electroporation. *Cold Spring Harb Protoc*. 2020;2020(6):pdb-prot101220. doi:10.1101/pdb.prot101220

15. Zimmermann L, Stephens A, Nam SZ, Rau D, Kübler J, Lozajic M, Gabler F, Söding J, Lupas AN, Alva V. A Completely Reimplemented MPI Bioinformatics Toolkit with a New HHpred Server at its Core. *J Mol Biol*. 2018;430(15):2237-2243. doi:10.1016/j.jmb.2017.12.007

16. Finn RD, Clements J, Eddy SR. HMMER web server: Interactive sequence similarity searching. *Nucleic Acids Res*. 2011;39(SUPPL. 2). doi:10.1093/nar/gkr367

17. Altschul SF, Madden TL, Schäffer AA, Zhang J, Zhang Z, Miller W, Lipman DJ. Gapped BLAST and PSI-BLAST: a new generation of protein database search programs. *Nucleic Acids Res*. 1997;25(17):3389-3402. doi:10.1093/nar/25.17.3389

18. Almagro Armenteros JJ, Tsirigos KD, Sønderby CK, Petersen TN, Winther O, Brunak S, von Heijne G, Nielsen H. SignalP 5.0 improves signal peptide predictions using deep neural networks. *Nat Biotechnol*. 2019;37(4):420-423. doi:10.1038/s41587-019-0036-z

19. Krogh A, Larsson B, Von Heijne G, Sonnhammer ELL. Predicting transmembrane protein topology with a hidden Markov model: Application to complete genomes. *J Mol Biol*. 2001;305(3):567-580. doi:10.1006/jmbi.2000.4315

20. Drula E, Garron ML, Dogan S, Lombard V, Henrissat B, Terrapon N. The carbohydrate-active enzyme database: Functions and literature. *Nucleic Acids Res*. 2022;50(D1):D571-D577. doi:10.1093/nar/gkab1045

21. Law J, Buist G, Haandrikman A, Kok J, Venema G, Leenhouts K. A. A system to generate chromosomal mutations in *Lactococcus lactis* which allows fast analysis of targeted genes. *J Bacteriol*. 1995;177(24):7011-7018. doi:10.1128/jb.177.24.7011-7018.1995

22. Álvarez-Martín P, Flórez AB, Margolles A, Del Solar G, Mayo B. Improved cloning vectors for bifidobacteria, based on the Bifidobacterium catenulatum pBC1 replicon. In: *Applied and Environmental Microbiology*. Vol 74. ; 2008:4656-4665. doi:10.1128/AEM.00074-08

23. Mckee LS, Peña MJ, Rogowski A, Jackson A, Lewis RJ, York WS, Krogh KBRM, Viksø-Nielsen A, Skjøt M, Gilbert HJ, et al. Introducing endo-xylanase activity into an exo-acting arabinofuranosidase that targets side chains. *Proceedings of the National Academy of Sciences*. 2012;109(17):6537-6542. doi:10.1073/pnas.1117686109/-/DCSupplemental

24. Rivière A, Moens F, Selak M, Maes D, Weckx S, De Vuyst L. The ability of bifidobacteria to degrade arabinoxylan oligosaccharide constituents and derived oligosaccharides is strain dependent. *Appl Environ Microbiol*. 2014;80(1):204-217. doi:10.1128/AEM.02853-13

25. Van Den Broek LAM, Lloyd RM, Beldman G, Verdoes JC, McCleary B V., Voragen AGJ. Cloning and characterization of arabinoxylan arabinofuranohydrolase-D3 (AXHd3) from *Bifidobacterium adolescentis* DSM20083. *Appl Microbiol Biotechnol*. 2005;67(5):641-647. doi:10.1007/s00253-004-1850-9

26. Santos CR, Polo CC, Costa MCMF, Nascimento AFZ, Meza AN, Cota J, Hoffmam ZB, Honorato R V., Oliveira PSL, Goldman GH, et al. Mechanistic strategies for catalysis adopted by evolutionary distinct family 43 arabinanases. *Journal of Biological Chemistry*. 2014;289(11):7362-7373. doi:10.1074/jbc.M113.537167

27. De Sanctis D, Inácio JM, Lindley PF, De Sá-Nogueira I, Bento I. New evidence for the role of calcium in the glycosidase reaction of GH43 arabinanases. *FEBS Journal*. 2010;277(21):4562-4574. doi:10.1111/j.1742-4658.2010.07870.x

28. Vandermarliere E, Bourgois TM, Winn MD, Van Campenhout S, Volckaert G, Delcour JA, Strelkov S V., Rabijns A, Courtin CM. Structural analysis of a glycoside hydrolase family 43 arabinoxylan arabinofuranohydrolase in complex with xylotetraose reveals a different binding mechanism compared with other members of the same family. *Biochemical Journal*. 2009;418(1):39-47. doi:10.1042/BJ20081256

29. Savard P, Roy D. Determination of Differentially Expressed Genes Involved in Arabinoxylan Degradation by *Bifidobacterium longum* NCC2705 Using Real-Time RT-PCR. *Probiotics Antimicrob Proteins*. 2009;1(2):121-129. doi:10.1007/s12602-009-9015-x

30. Gosalbes MJ, Pérez-González JA, Gonzalez R, Navarro A. Two beta-glycanase genes are clustered in *Bacillus polymyxa*: molecular cloning, expression, and sequence analysis of genes encoding a xylanase and an endo-beta-(1, 3)-(1, 4)-glucanase. *J Bacteriol*. 1991;173(23):7705-7710. doi:10.1128/jb.173.23.7705-7710.1991
